# Clinical-metabolic machine learning model differentiating rheumatoid arthritis from high-inflammatory Sjögren disease: A multi-center study

**DOI:** 10.1016/j.isci.2026.117299

**Published:** 2026-08-24

**Authors:** Jianbin Li, Suiran Li, Renhe Li, Ning Tan, Xiaoqing Wang, Mengxia Liu, Yuzhen Gesang, Wei Liu

**Affiliations:** 1 Department of Rheumatism and Immunity, First Teaching Hospital of Tianjin University of Traditional Chinese Medicine, Tianjin, China; 2 National Clinical Research Center for Chinese Medicine, Tianjin, China; 3 Shenzhen Nanshan People’s Hospital, Shenzhen, China; 4 Henan Provincial Orthopedic Hospital, Henan, China; 5 Department of Rheumatology and Immunology, The First Affiliated Hospital, Jiangxi Medical College, Nanchang University, Nanchang, China

**Keywords:** rheumatoid arthritis, Sjögren’s syndrome, machine learning, XGBoost, clinical-metabolic combined model, SHAP, differential diagnosis, high-RF phenotype

## Abstract

Although rheumatoid arthritis (RA) and the high-inflammatory phenotype of Sjögren disease (SjD-Phenotype 1) share clinical features including RF positivity and articular involvement, their differentiation remains challenging, particularly in anti-CCP-negative or indeterminate cases. Here, we developed and validated a machine learning model based on routine metabolic biomarkers to distinguish these two conditions. In the discovery cohort (Tianjin, *n* = 5,330: 4,736 RA and 594 SjD-Phenotype 1), XGBoost modeling was performed after excluding underlying liver diseases, with glucocorticoids (GCs) and hydroxychloroquine (HCQ) incorporated as key covariates. The model achieved an AUC of 0.88 (95% CI: 0.84–0.91) on the held-out test set, with 5-fold cross-validation confirming robust calibration (mean Brier score = 0.141, calibration slope = 1.03). Exploratory analysis in the ACPA-negative subgroup yielded an AUC of 0.910, though this estimate includes training samples and should be interpreted with caution. External validation in an independent Nanchang cohort (*n* = 319: 236 RA, 83 SjD) confirmed generalizability, yielding a pooled AUC of 0.840 (95% CI: 0.791–0.890; Rubin’s rules) with consistent SHAP feature importance rankings. Model ablation analysis confirmed that while glucocorticoid use was the strongest individual discriminating feature, metabolic features provided significant incremental value beyond treatment variables alone (ΔAUC = +0.086, DeLong *p* < 0.001). Multivariable analysis demonstrated that elevated glucose (OR = 1.20, *p* = 0.028) and CRP (OR = 2.54, *p* < 0.001) remained independent discriminating features for RA after dual adjustment for GC and HCQ use. To explore the molecular basis of this metabolic divergence, we performed parallel transcriptomic analyses of publicly available PBMC datasets (GSE51092 for pSS, GSE93272 for RA), revealing fundamentally distinct pathway signatures: RA exhibited dominant mitochondrial oxidative phosphorylation and ribosomal gene programs, while SjD was characterized by robust type I interferon activation—providing molecular-level corroboration for the clinically observed metabolic differences. These findings demonstrate that routine biochemical parameters can provide auxiliary diagnostic value in the serological gray zone where anti-CCP fails, supported by both multi-center clinical validation and transcriptomic biological evidence.

## Highlights

• ML model distinguishes RA from high-inflammatory SjD using metabolic biomarkers
• Metabolic features add significant value beyond treatment patterns (ΔAUC = +0.086)
• Glucose and CRP remain independent RA discriminators after GC/HCQ adjustment
• Transcriptomic analysis confirms divergent metabolic pathways in RA vs. SjD

## Graphical abstract

**Figure:**
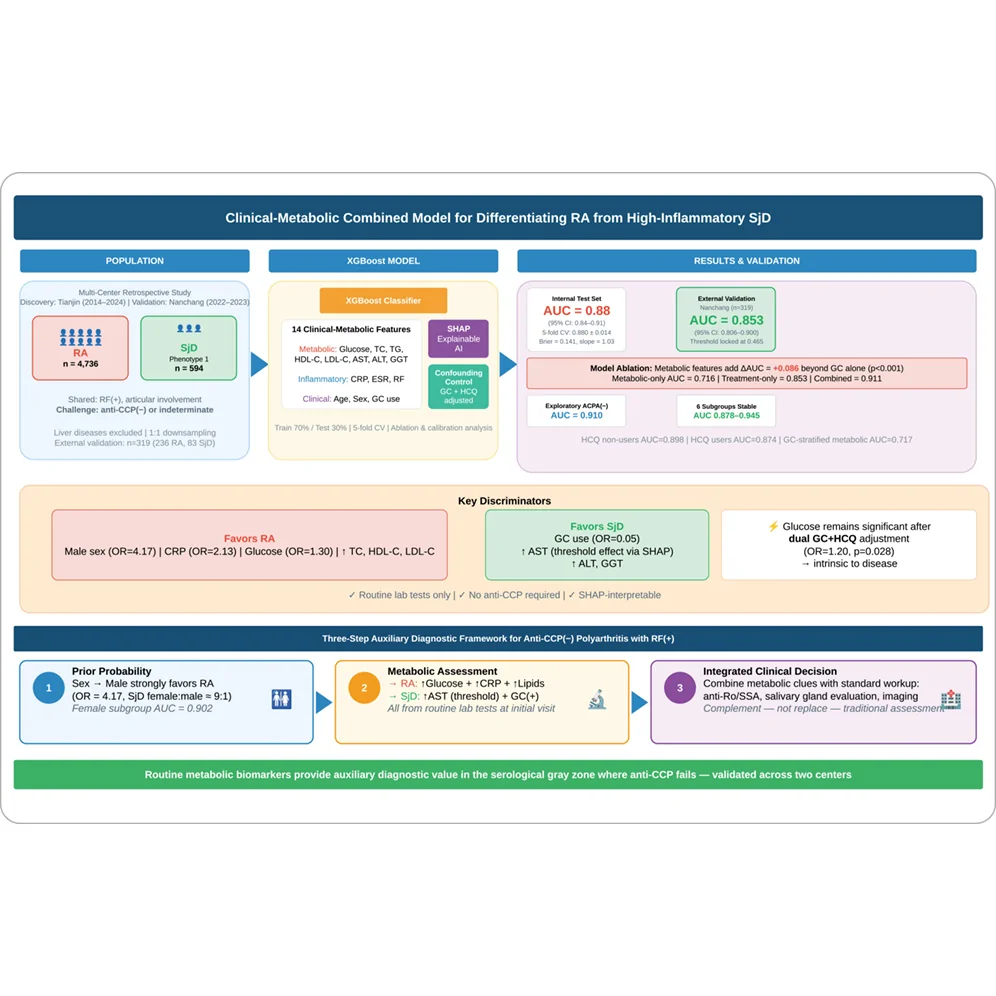

## Teaser

Computer-aided diagnosis method; Machine learning; Rheumatology

## Introduction

Rheumatoid arthritis (RA) and Sjögren disease (SjD) are among the most common systemic autoimmune diseases, affecting millions worldwide and imposing significant disease burden and healthcare expenditure.^1^^,^^2^ Although traditionally considered distinct clinical entities, these two diseases share multiple immunological features, including rheumatoid factor (RF) positivity, systemic inflammation, and articular manifestations, posing considerable challenges for differential diagnosis in clinical practice.^3^^,^^4^

The clinical heterogeneity of SjD has been increasingly recognized. Recent cluster analysis studies have identified distinct SjD phenotypes, among which a high-inflammatory articular phenotype (hereinafter referred to as Phenotype 1) is characterized by high RF titers, elevated inflammatory markers, and prominent musculoskeletal involvement, showing substantial clinical overlap with RA.^5^^,^^6^ Our previous study using unsupervised machine learning identified this inflammatory subgroup (Phenotype 1), which has a 100% joint involvement rate and significantly elevated RF levels, making it particularly challenging to differentiate from RA.^7^ Notably, because this subgroup is defined by high RF titers, RF itself has *a priori* lost its discriminative value in this clinical context—this is precisely why we urgently need to identify alternative biomarkers.

Current diagnostic approaches rely primarily on disease-specific autoantibodies, namely anti-cyclic citrullinated peptide (anti-CCP) antibodies for RA and anti-Ro/SSA and anti-La/SSB antibodies for SjD.^8^^,^^9^ However, these markers have limitations: anti-CCP has a sensitivity of 61–75%, and some SjD patients (particularly the inflammatory phenotype) may present with borderline or negative results, especially in early disease stages.^10^^,^^11^ Notably, anti-Ro/SSA antibodies can also be present in some RA patients, and this serological overlap further increases diagnostic difficulty. Additionally, RF, a historical core indicator for RA diagnosis, is also positive in 40–50% of SjD patients, particularly those with extraglandular manifestations.^12^ This study specifically focuses on the clinical gray zone where anti-CCP is negative or indeterminate, where clinicians cannot rely on anti-CCP for rapid definitive diagnosis and urgently need other auxiliary markers.

Emerging evidence suggests that RA and SjD may exhibit distinct metabolic profiles beyond autoantibody characteristics. RA has been established to be associated with accelerated atherosclerosis, insulin resistance, and dyslipidemia, driven by chronic systemic inflammation and metabolic reprogramming of immune cells.^13^^,^^14^ In contrast, the pathogenesis of SjD centers on B-cell hyperactivation and lymphocytic infiltration of exocrine glands, potentially producing different metabolic consequences.^15^^,^^16^ At the molecular level, these distinct pathophysiological pathways engage fundamentally different metabolic programs: RA synovial immune cells undergo metabolic reprogramming toward aerobic glycolysis, whereas SjD pathogenesis is driven primarily by type I interferon-mediated immune activation.^17^^,^^18^ These differences suggest that routine metabolic and biochemical parameters may harbor unexplored differential diagnostic value. Machine learning methods provide powerful tools for identifying complex patterns in high-dimensional clinical data and have shown promise in autoimmune disease differential diagnosis and phenotyping.^19^^,^^20^ Importantly, the clinical utility of such models depends not only on their predictive performance but also on feature interpretability, enabling clinicians to understand which variables drive classification decisions.^21^ Recent advances in explainable AI, particularly SHapley Additive exPlanations (SHAP) analysis, provide reliable methods for interpreting complex models while maintaining their predictive power.

This study aimed to develop and validate a machine learning model based on demographic characteristics, inflammatory status, and routine metabolic biomarkers to differentiate RA from the high-inflammatory articular phenotype of SjD. We hypothesized that despite similar clinical presentations, these two diseases possess distinct clinical and metabolic characteristics that can complement traditional diagnostic approaches, especially by providing auxiliary diagnostic evidence for anti-CCP-negative or indeterminate patients. To enhance the robustness and clinical applicability of our findings, we performed external validation in an independent cohort from a geographically distinct center and conducted transcriptomic pathway analysis using publicly available datasets to provide molecular-level biological corroboration for the observed clinical metabolic divergence.

## Results

### Baseline characteristics

A total of 5,330 patients were enrolled in the discovery cohort from Tianjin: 4,736 RA and 594 SjD-Phenotype 1. Baseline characteristics are presented in Table 1. RA patients were significantly older (62.9 ± 11.2 vs. 57.4 ± 12.4 years, *p* < 0.001), with a higher proportion of males (21.5% vs. 6.4%, *p* < 0.001). The RA cohort had a significantly higher comorbidity burden, with higher prevalence of hypertension (35.5% vs. 10.9%, *p* < 0.001) and diabetes (12.1% vs. 1.5%, *p* < 0.001). The two groups showed significant differences in inflammatory characteristics. RA patients had higher CRP levels (25.2 ± 38.0 vs. 15.3 ± 23.4 mg/L, *p* < 0.001), while ESR levels were comparable (49.5 ± 30.6 vs. 51.2 ± 29.7 mm/h, *p* = 0.112). SjD-Phenotype 1 patients had higher mean RF titers (216.7 ± 1090.1 vs. 186.0 ± 485.7 IU/mL, *p* < 0.001), but with markedly greater variability. This RF distribution pattern conforms to the study’s enrollment design: SjD-Phenotype 1 is defined as the high-RF subgroup; therefore, high RF levels are a defining characteristic rather than a discovery. Metabolic parameters showed distinct patterns. RA patients had higher fasting glucose (5.37 ± 1.73 vs. 4.92 ± 1.20 mmol/L, *p* < 0.001) and a more atherogenic lipid profile, with elevated total cholesterol (TC) (4.65 ± 1.24 vs. 4.37 ± 1.10 mmol/L), high-density lipoprotein cholesterol (HDL-C) (1.15 ± 0.33 vs. 1.10 ± 0.32 mmol/L), and low-density lipoprotein cholesterol (LDL-C) (2.85 ± 0.89 vs. 2.63 ± 0.82 mmol/L) (all *p* < 0.001). Conversely, SjD-Phenotype 1 patients had significantly higher liver enzymes: AST (25.3 ± 28.7 vs. 19.3 ± 18.7 U/L, *p* < 0.001) and ALT (23.5 ± 31.8 vs. 18.1 ± 21.6 U/L, *p* < 0.001). Regarding medication use, the two groups showed significant differences in treatment regimens. Glucocorticoid (GC) usage was significantly higher in the SjD-Phenotype 1 group compared to the RA group (71.9% vs. 13.7%, *p* < 0.001). Additionally, hydroxychloroquine (HCQ) usage rates differed significantly between groups: SjD-Phenotype 1 was significantly higher than RA (43.4% vs. 3.4%, *p* < 0.001), consistent with clinical practice for HCQ in SjD treatment. Figure S1 displays violin plots showing the distribution differences of AST and glucose—two key metabolic features—between groups. After Bonferroni correction (17 comparisons, adjusted significance threshold: *p* < 0.00294), 14 of 17 variables remained statistically significant. Only ESR (*p* = 0.112), TG (*p* = 0.877), and GGT (*p* = 0.074) did not reach significance, consistent with their limited value in differentiating these two diseases.

**Table 1:** Baseline characteristics of RA and SjD-phenotype 1 (high-inflammatory articular phenotype) patients—discovery cohort (Tianjin)

Characteristic	RA (*n* = 4736)	SjD-phenotype 1 (*n* = 594)	*p* value
Age (years)	62.85 ± 11.23	57.41 ± 12.36	<0.001∗
Male gender, *n* (%)	1019 (21.5%)	38 (6.4%)	<0.001∗
Comorbidities			
Hypertension, *n* (%)	1681 (35.5%)	65 (10.9%)	<0.001∗
Diabetes, *n* (%)	574 (12.1%)	9 (1.5%)	<0.001∗
Inflammatory markers			
ESR (mm/h)	49.48 ± 30.61	51.15 ± 29.74	0.112
CRP (mg/L)	25.22 ± 37.96	15.29 ± 23.37	<0.001∗
Serology			
RF (IU/mL)	186.04 ± 485.73	216.74 ± 1090.05	<0.001∗
Metabolic parameters			
Glucose (mmol/L)	5.37 ± 1.73	4.92 ± 1.20	<0.001∗
TC (mmol/L)	4.65 ± 1.24	4.37 ± 1.10	<0.001∗
TG (mmol/L)	1.31 ± 0.87	1.32 ± 0.79	0.877
HDL-C (mmol/L)	1.15 ± 0.33	1.10 ± 0.32	<0.001∗
LDL-C (mmol/L)	2.85 ± 0.89	2.63 ± 0.82	<0.001∗
Liver enzymes			
ALT (U/L)	18.09 ± 21.56	23.49 ± 31.80	<0.001∗
AST (U/L)	19.32 ± 18.68	25.26 ± 28.69	<0.001∗
GGT (U/L)	31.00 ± 41.32	31.41 ± 52.83	0.074
Medication use			
HCQ use, *n* (%)	159 (3.4%)	258 (43.4%)	<0.001∗
Glucocorticoid use, *n* (%)	648 (13.7%)	427 (71.9%)	<0.001∗

Continuous variables are presented as mean ± standard deviation; categorical variables as *n* (%). Wilcoxon rank-sum test was used for continuous variables; chi-square test for categorical variables. ∗Indicates statistical significance after Bonferroni correction for multiple comparisons (adjusted threshold: *p* < 0.05/17 = 0.00294). Of 17 comparisons, 14 remained significant after correction; ESR, TG, and GGT did not reach significance after correction.

RA, rheumatoid arthritis; SjD, Sjögren disease; ESR, erythrocyte sedimentation rate; CRP, C-reactive protein; RF, rheumatoid factor; TC, total cholesterol; TG, triglycerides; HDL-C, high-density lipoprotein cholesterol; LDL-C, low-density lipoprotein cholesterol; ALT, alanine aminotransferase; AST, aspartate aminotransferase; GGT, γ-glutamyl transferase; HCQ, hydroxychloroquine.

### Machine learning model performance

**Figure 1:**
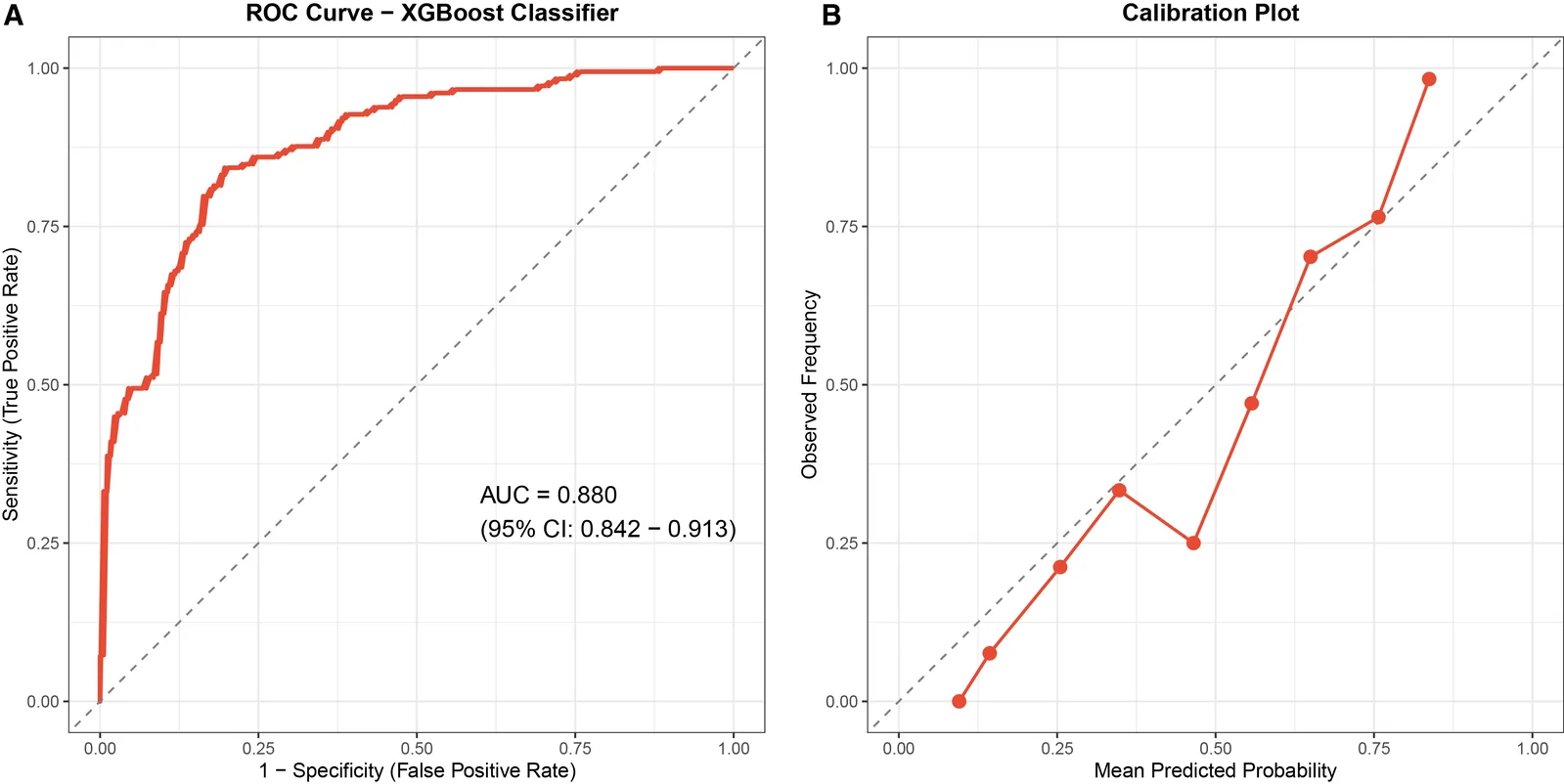
Model performance evaluation—discovery cohort (A) Receiver operating characteristic curve for the XGBoost classifier (AUC = 0.88, 95% CI: 0.84–0.91). (B) Calibration plot shows agreement between predicted probabilities and observed frequencies, demonstrating good calibration performance.

After 1:1 downsampling, the balanced dataset contained 1,188 patients (594 per group). The XGBoost classifier demonstrated excellent discriminative ability on the independent test set, achieving an AUC of 0.88 (95% CI: 0.84–0.91) on the held-out test set (30% of the balanced 1:1 downsampled dataset, *n* = 356) (Figure 1A). To avoid optimistic bias from evaluating the model on data that included training samples, we report the held-out test set AUC (0.88) as the primary performance metric throughout. The subgroup AUCs in Table S6 were calculated by applying the trained model to the full unbalanced cohort; as these include training samples, they may be optimistically biased and are reported for exploratory subgroup comparison only. This performance significantly exceeds expectations based solely on traditional serological markers, highlighting the discriminative value of multidimensional clinical-metabolic features. 5-fold cross-validation further confirmed model robustness, with a mean AUC of 0.879 (SD = 0.012), indicating absence of overfitting. Cross-validation also demonstrated good calibration (mean Brier score = 0.141 ± 0.009, calibration intercept = −0.001 ± 0.140, calibration slope = 1.028 ± 0.095; Table S4), indicating reliable probability estimates. Multi-model comparison showed that XGBoost performed comparably to multivariable logistic regression (AUC = 0.87), both effectively capturing inter-group feature differences. The calibration plot (Figure 1B) showed good agreement between predicted probabilities and observed frequencies, indicating high reliability of the model’s probability estimates. UMAP dimensionality reduction visualization (Figure S2) further corroborated these results, showing that RA and SjD-Phenotype 1 exhibit clearer separation patterns in the high-dimensional space combining GC use and metabolic features.

### Independent discriminating features

**Figure 2:**
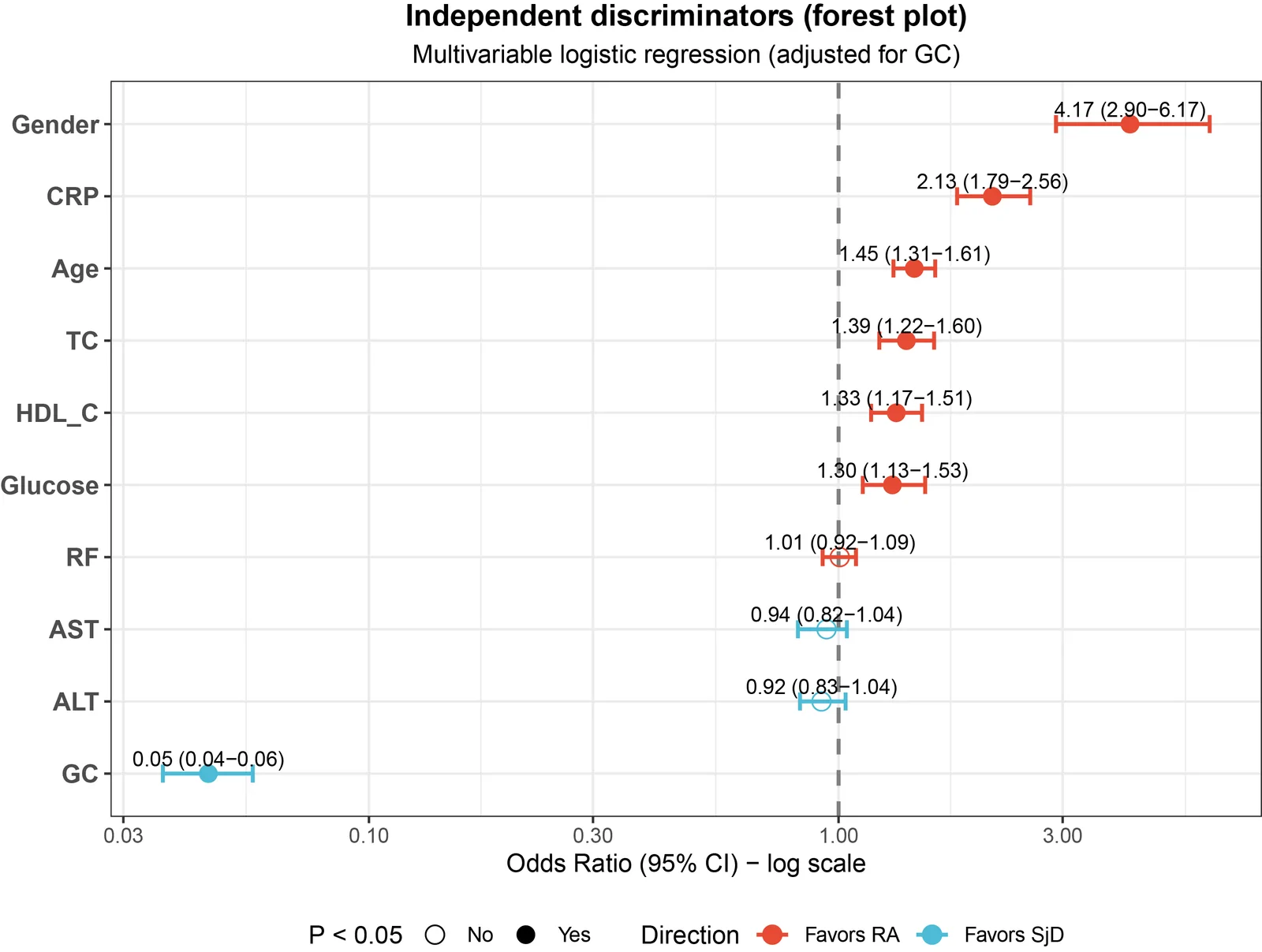
Independent discriminating features identified by multivariable logistic regression Forest plot shows standardized odds ratios and 95% confidence intervals. Variables with OR > 1 favor RA classification; variables with OR < 1 favor SjD-Phenotype 1 classification. Solid circles indicate statistical significance (*p* < 0.05).

Multivariable logistic regression analysis of standardized variables identified multiple independent discriminating factors (Figure 2 and Table 2). The strongest predictor for RA classification was male sex (OR = 4.17, 95% CI: 2.90–6.17, *p* < 0.001). This result reflects the epidemiological characteristic that SjD is extremely female-predominant (female:male approximately 9:1 or higher), while RA, although also female-predominant, has a relatively higher male proportion. This sex difference is an inherent characteristic of disease epidemiology and has practical reference value in clinical practice. Among metabolic variables, even with rigorous adjustment incorporating GCs as a covariate, elevated CRP (OR = 2.13, 95% CI: 1.79–2.56, *p* < 0.001) and elevated glucose (OR = 1.30, 95% CI: 1.13–1.53, *p* < 0.001) remained independent predictors for RA. This is consistent with the interpretation that the hyperglycemic profile in RA is not solely caused by GC therapy but reflects intrinsic metabolic dysregulation of the disease. Additionally, older age (OR = 1.45), elevated TC (OR = 1.39), and elevated HDL-C (OR = 1.33) also independently favored RA classification. Notably, AST no longer showed statistically significant independent discriminative value after adjustment for GC use (OR = 0.94, 95% CI: 0.82–1.04, *p* = 0.325). This suggests that the liver enzyme differences observed in univariate analysis may be partially confounded by treatment background (such as GC use) rather than being completely independent disease characteristics. Similarly, ALT did not show independent discriminative value (*p* = 0.129). GC use showed an extremely strong classification effect (OR = 0.05, 95% CI: 0.04–0.06, *p* < 0.001), strongly favoring SjD-Phenotype 1 classification, reflecting the extremely high usage rate of GCs in treating this SjD subgroup. As anticipated by the study design, RF did not show significant independent discriminative value (OR = 1.01, 95% CI: 0.92–1.09, *p* = 0.882). This result conforms to design expectations: the defining characteristic of SjD-Phenotype 1 is high RF titers; therefore, RF’s loss of discriminative ability between these specific subgroups is logical. This is precisely the premise and starting point for our search for alternative markers.

**Table 2:** Multivariable logistic regression analysis of independent discriminating features

Variable	OR (95% CI)	*p* value	Direction
Gender (Male)	4.17 (2.90–6.17)	<0.001	favors RA
CRP	2.13 (1.79–2.56)	<0.001	favors RA
Glucose	1.30 (1.13–1.53)	<0.001	favors RA
Age	1.45 (1.31–1.61)	<0.001	favors RA
HDL-C	1.33 (1.17–1.51)	<0.001	favors RA
TC	1.39 (1.22–1.60)	<0.001	favors RA
RF	1.01 (0.92–1.09)	0.882	–
ALT	0.92 (0.83–1.04)	0.129	–
AST	0.94 (0.82–1.04)	0.325	–
Glucocorticoids (GC)	0.05 (0.04–0.06)	<0.001	favors SjD

OR > 1 indicates the variable favors RA classification; OR < 1 indicates the variable favors SjD-phenotype 1 classification. Continuous variables were *z* score standardized. Bold indicates statistical significance (*p* < 0.05). This model was adjusted for glucocorticoid (GC) use; HCQ was not included as a covariate. For dual GC + HCQ adjustment results, see Table S1.

OR, odds ratio; CI, confidence interval; AUC, area under the receiver operating characteristic curve; RA, rheumatoid arthritis; SjD, Sjögren disease; RF, rheumatoid factor; CRP, C-reactive protein; TC, total cholesterol; HDL-C, high-density lipoprotein cholesterol; AST, aspartate aminotransferase; ALT, alanine aminotransferase; GC, glucocorticoids.

### SHAP feature importance analysis

**Figure 3:**
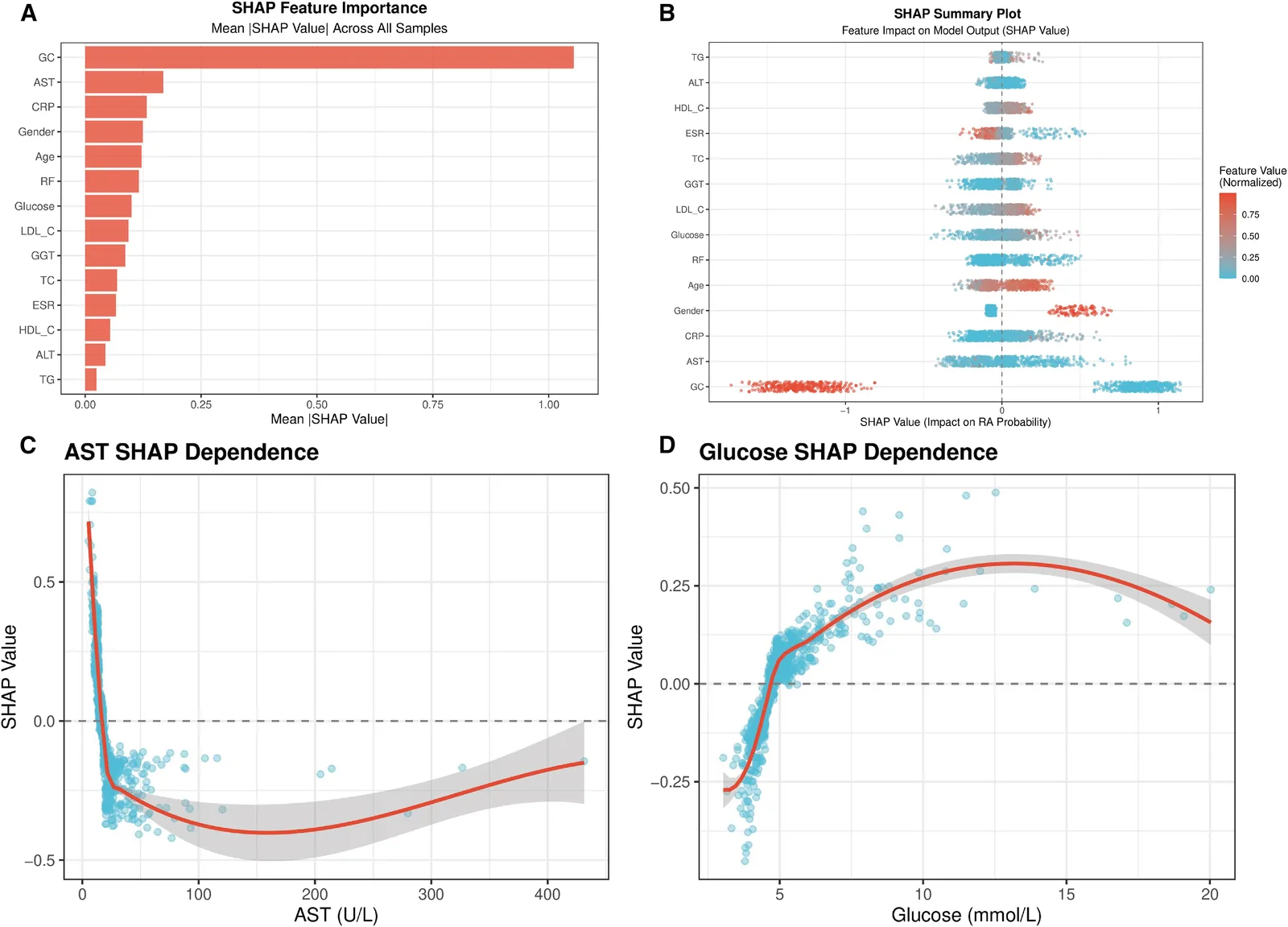
SHAP analysis revealing feature contributions to model predictions (A) SHAP feature importance plot. Glucocorticoids (GC, 1.054) is the feature contributing most to model predictions, followed by AST (0.169), CRP (0.133), sex (0.124), and age (0.121). (B) SHAP summary plot. Red indicates high feature values; blue indicates low feature values. (C and D) SHAP dependence plots revealing non-linear effects of key features. (C) The AST SHAP dependence plot shows a clear non-linear threshold effect. (D) The glucose SHAP dependence plot shows a positive non-linear relationship with RA classification.

SHAP analysis provided mechanistic insights into XGBoost model predictions (Figure 3). Consistent with logistic regression results, GCs ranked first in SHAP feature importance, with a mean absolute SHAP value of 1.054, confirming their decisive influence on classification discrimination. Notably, although AST lost independent significance in multivariable logistic regression, it ranked second in SHAP analysis (0.169), above CRP (0.133), sex (0.124), and age (0.121). This discrepancy highlights machine learning’s advantage in capturing non-linear relationships between variables. The dependence plot in Figure 3C reveals the reason: AST’s contribution to the model is not a simple linear relationship but exhibits a clear threshold effect—it significantly increases the probability of SjD classification only within specific elevated ranges. Such complex non-linear patterns are easily obscured in traditional linear regression. Furthermore, CRP, as a representative inflammatory indicator, ranked third (0.133). The SHAP summary plot (Figure 3B) clearly demonstrates each feature’s directional contribution: higher CRP values produce positive SHAP values, pushing the model toward RA classification; while higher AST values and GC use produce negative SHAP values, strongly indicating SjD classification. This aligns highly with the aforementioned clinical findings.

### Subgroup analysis

Model performance maintained excellent stability across all clinically relevant subgroups (Table S6). Stratification by RF level showed that the model demonstrated strong discriminative ability in both the low-titer group (RF ≤ 200 IU/mL: AUC = 0.918) and the most challenging high-titer group (RF > 200 IU/mL: AUC = 0.878), confirming its robustness independent of serological status. Age stratification analysis showed comparable prediction accuracy between younger (AUC = 0.922) and older patients (AUC = 0.895), indicating that the model is not affected by age-related natural metabolic fluctuations. Sex stratification analysis results were particularly encouraging. Although the male subgroup showed extremely high AUC (0.945), in the female subgroup—which has the largest sample size and greatest clinical diagnostic difficulty—the model still achieved an excellent AUC of 0.902. Because SjD is a typically female-predominant disease, the high accuracy in the female subgroup powerfully demonstrates that the model’s predictive ability does not simply rely on sex as a demographic difference, but successfully captures deeper metabolic and treatment background differences between groups. Additionally, AUC was as high as 0.910 in the non-diabetic population; however, because SjD-Phenotype 1 patients with comorbid diabetes were extremely rare (*n* = 9), reliable statistics could not be performed for this specific subgroup, suggesting that clinical interpretation should be context-dependent for patients with comorbid diabetes.

### Exploratory anti-CCP negative subgroup analysis

**Figure 4:**
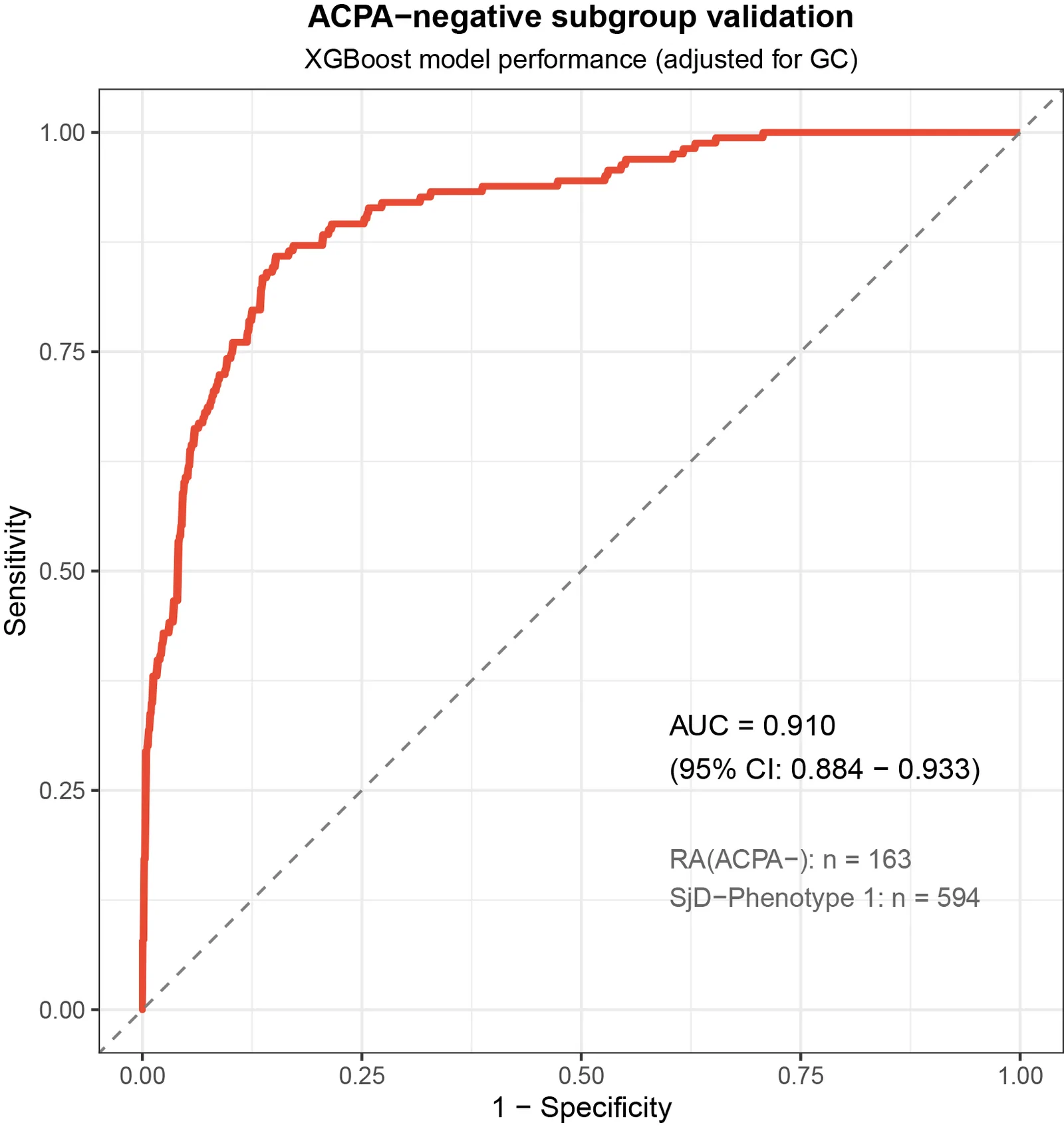
Receiver operating characteristic curve for validation in the anti-CCP negative RA subgroup In the comparison between ACPA-negative RA (*n* = 163) and SjD-Phenotype 1 (*n* = 594), the XGBoost model yielded an AUC of 0.910 (95% CI: 0.884–0.933)

As an exploratory analysis, we applied the trained model to the comparison between ACPA-negative RA (*n* = 163) and SjD-Phenotype 1 (*n* = 594). The model yielded an AUC of 0.910 (95% CI: 0.884–0.933) in this subgroup (Figure 4). As this analysis includes training samples, the AUC estimate may be optimistically biased; prospective validation in a fully held-out ACPA-negative cohort is warranted. The consistency of this exploratory AUC with the full-cohort estimate (0.911, Table S6)—both calculated using the same evaluation method on unbalanced data—suggests that model performance was not diminished in serologically atypical patients, though this finding requires confirmation in an independent held-out cohort. This performance was highly consistent with the main analysis of the full cohort (AUC = 0.911), indicating that the model’s predictive ability was not diminished by the absence of specific serological antibodies (ACPA). This validation analysis directly confirms the model’s robustness and clinical utility in serologically atypical cases—the target application population of this study. Baseline characteristics of the ACPA-negative subgroup (Table 3) showed that compared to the SjD-Phenotype 1 group, ACPA-negative RA patients had higher CRP levels (22.4 ± 35.4 vs. 15.3 ± 23.4 mg/L), though the difference did not reach statistical significance (*p* = 0.078). However, the inter-group metabolic feature difference patterns remained highly consistent with and significant as in the main analysis: ACPA-negative RA patients had significantly higher glucose (5.43 ± 1.73 vs. 4.92 ± 1.20 mmol/L, *p* < 0.001), TC, HDL-C, and LDL-C levels, as well as significantly lower AST levels (19.5 ± 9.7 vs. 25.3 ± 28.7 U/L, *p* < 0.001). This stable metabolic difference pattern in the serology-negative population powerfully supports the biological basis for metabolic features as independent discriminating markers.

**Table 3:** Baseline characteristics of ACPA-negative RA subgroup vs. SjD-phenotype 1

Characteristic	RA_ACPA- (*n* = 163)	SjD-phenotype 1 (*n* = 594)	*p* value
Age	61.44 ± 11.82	57.41 ± 12.36	<0.001
ESR	35.44 ± 30.37	51.15 ± 29.74	<0.001
CRP	22.39 ± 35.42	15.29 ± 23.37	0.078
RF	44.49 ± 166.31	216.74 ± 1090.05	<0.001
TC	4.85 ± 1.13	4.37 ± 1.10	<0.001
TG	1.39 ± 0.80	1.32 ± 0.79	0.092
HDL-C	1.24 ± 0.32	1.10 ± 0.32	<0.001
LDL-C	3.01 ± 0.89	2.63 ± 0.82	<0.001
Glucose	5.43 ± 1.73	4.92 ± 1.20	<0.001
ALT	19.11 ± 12.04	23.49 ± 31.80	0.711
AST	19.52 ± 9.73	25.26 ± 28.69	<0.001
GGT	36.92 ± 61.45	31.41 ± 52.83	0.019
Gender (male), *n* (%)	39 (23.9%)	38 (6.4%)	<0.001
Hypertension, *n* (%)	46 (28.2%)	65 (10.9%)	<0.001
Diabetes, *n* (%)	19 (11.7%)	9 (1.5%)	<0.001
HCQ, *n* (%)	7 (4.3%)	258 (43.4%)	<0.001
Glucocorticoid use, *n* (%)	21 (12.9%)	427 (71.9%)	<0.001

### Sensitivity analysis

**Figure 5:**
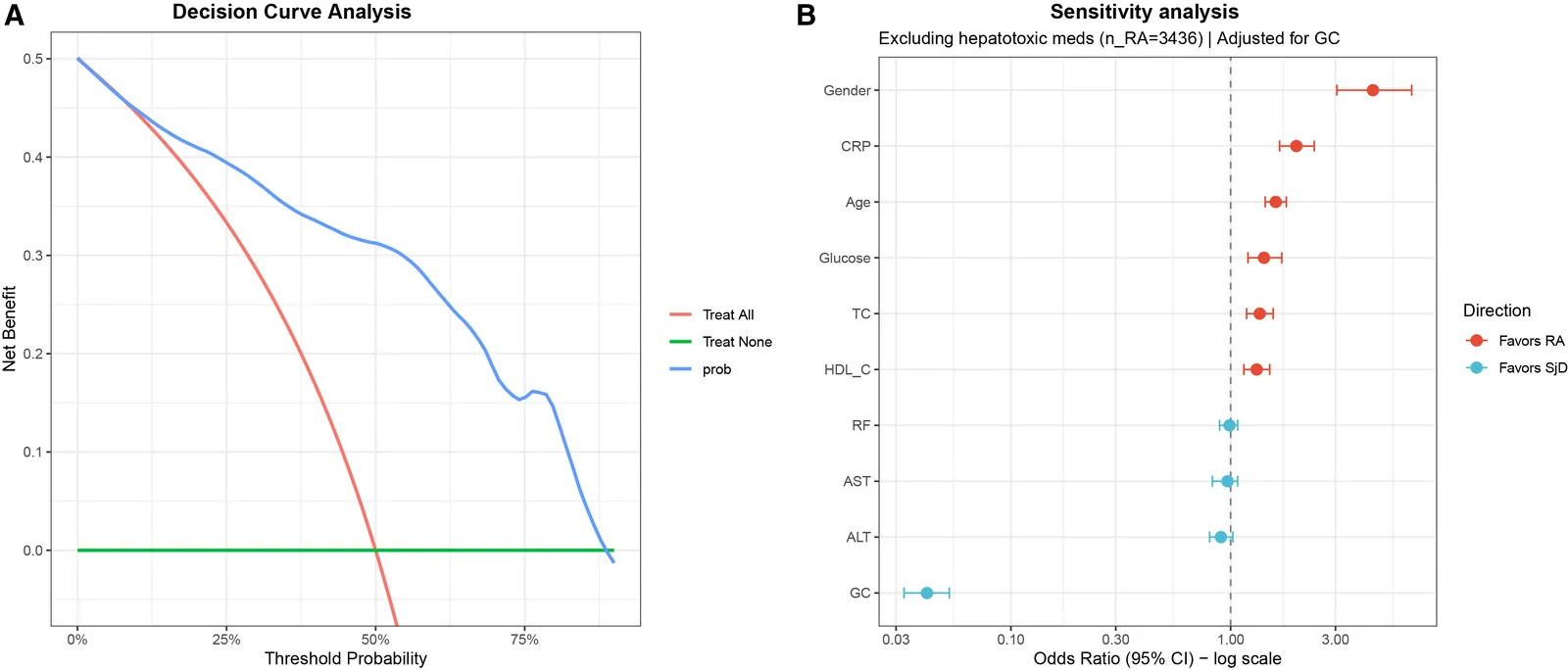
Assessment of clinical utility and model robustness (A) Decision curve analysis (DCA). The decision curve shows the net benefit of the XGBoost model (red line) compared to treating all patients (gray line) or treating none (black line). The model demonstrates positive net benefit across a wide threshold probability range (10%–85%). (B) Sensitivity analysis. Forest plot shows multivariable logistic regression results in the subgroup of RA patients naive to methotrexate and leflunomide (*n* = 3,436) versus SjD-Phenotype 1 (*n* = 594).

To exclude potential confounding effects of disease-modifying antirheumatic drugs (DMARDs) on liver function, we performed a sensitivity analysis on a subgroup of 3,436 RA patients naive to methotrexate or leflunomide. After rigorous adjustment incorporating GCs, analysis results remained highly consistent with the main full-cohort analysis (Figure 5B): Glucose remained a robust independent discriminating feature (OR = 1.42, 95% CI: 1.20–1.71), favoring RA classification; while AST did not show independent significance after medication background adjustment (OR = 0.97, 95% CI: 0.83–1.08). This finding powerfully demonstrates that the observed metabolic differences (particularly hyperglycemia) reflect the intrinsic biological characteristics of the disease rather than being solely induced by hepatotoxic medications.

Decision curve analysis (DCA, Figure 5A) showed that across a broad threshold probability range of 10%–85%, the clinical-metabolic combined model provided significant net clinical benefit compared to “treat all” or “treat none” strategies. This means that using this model to assist decision-making can bring practical benefits to patients in the vast majority of clinical diagnostic uncertainty scenarios.

### Hydroxychloroquine confounding analysis

Given the significantly higher HCQ usage in the SjD-Phenotype 1 group (43.4% vs. 3.4%) and the known glucose-lowering and lipid-improving effects of HCQ, we performed comprehensive analyses to evaluate whether HCQ confounded the observed metabolic differences. In the multivariable logistic regression model simultaneously adjusting for both GC and HCQ, glucose remained a statistically significant independent discriminator for RA (OR = 1.20, 95% CI: 1.02–1.41, *p* = 0.028), as did CRP (OR = 2.54, *p* < 0.001) and HDL-C (OR = 1.34, *p* = 0.001). The attenuation of the glucose OR from 1.30 (GC-only adjustment) to 1.20 (GC + HCQ adjustment) was modest, confirming that the hyperglycemic profile in RA is predominantly driven by intrinsic disease biology rather than differential HCQ exposure. HCQ itself emerged as a strong classifier favoring SjD-Phenotype 1 (OR = 0.08, *p* < 0.001), comparable in magnitude to GCs. HCQ-stratified analysis further corroborated these findings. Among HCQ non-users (*n* = 4,913), RA patients had significantly higher glucose than SjD-Phenotype 1 patients (5.38 ± 1.74 vs. 4.93 ± 1.19 mmol/L, *p* < 0.001), along with significantly higher TC (*p* < 0.001), LDL-C (*p* < 0.001), and significantly lower AST (19.2 ± 18.7 vs. 24.8 ± 28.6 U/L, *p* < 0.001). Among HCQ users (*n* = 417), AST remained significantly higher in the SjD group (*p* < 0.001), while glucose differences were attenuated and non-significant (5.00 ± 1.15 vs. 4.90 ± 1.21, *p* = 0.200), likely attributable to both the small RA sample (*n* = 159) and the glucose-lowering effect of HCQ. The XGBoost model maintained robust discriminative performance in both strata: AUC = 0.898 (95% CI: 0.880–0.915) for HCQ non-users and AUC = 0.874 (95% CI: 0.841–0.908) for HCQ users. These results demonstrate that the model’s classification ability is not dependent on HCQ-related metabolic confounding (Table S1).

### Model ablation and feature category analysis

To assess the contribution of treatment variables to model performance, we performed systematic ablation and feature category analyses (Figures S4 and S5 and Tables S2 and S3). For the ablation analysis, a 15-feature model including both GC and HCQ served as the reference (AUC = 0.911, 95% CI: 0.881–0.940; note that this AUC is higher than the primary 14-feature model AUC of 0.88, reflecting the additional discriminative contribution of HCQ). Removing GC reduced AUC to 0.829 (ΔAUC = −0.082, DeLong *p* < 0.001), confirming GC as the strongest individual contributor. Removing HCQ had a smaller but significant effect (AUC = 0.879, ΔAUC = −0.032, *p* < 0.001), and removing both treatment variables yielded an AUC of 0.716 (ΔAUC = −0.195, *p* < 0.001).

Feature category analysis further dissected these contributions (Table S3). A treatment-only model (GC + HCQ, 2 features) achieved AUC = 0.853 (95% CI: 0.816–0.889), while a metabolic-only model (13 clinical features excluding treatment variables) achieved AUC = 0.716 (95% CI: 0.663–0.768). The combined model (AUC = 0.911) significantly outperformed both (all DeLong *p* < 0.001). Importantly, incremental analysis demonstrated that adding metabolic features to GC alone significantly improved discrimination (GC-only AUC = 0.792 vs. GC + metabolic AUC = 0.879, ΔAUC = +0.086, DeLong *p* < 0.001), confirming that metabolic features capture independent biological information beyond treatment patterns (Table S5).

To further control for potential treatment confounding, propensity score matching on GC use (equalizing GC rates between groups at 31.8%) confirmed that the metabolic-only model retained meaningful discriminative ability (AUC = 0.698, 95% CI: 0.614–0.783). GC-stratified analyses showed that among GC non-users—where treatment background is identical—the metabolic-only model still achieved AUC = 0.717 (95% CI: 0.637–0.797), supporting the presence of genuine biological metabolic differences independent of treatment effects (Table S5).

### External validation in an independent cohort

To address the critical need for external validation, we independently tested the trained XGBoost model on a geographically distinct cohort from the First Affiliated Hospital of Nanchang University (*n* = 319: 236 RA, 83 SjD-Phenotype 1). This external cohort differed from the discovery cohort in several important respects (Table 4): patients were younger (mean age 53.6 vs. 62.9 years), had lower CRP levels (mean 11.9 vs. 25.2 mg/L), and showed different medication patterns, reflecting real-world inter-center variability.

**Table 4:** Baseline characteristics of the external validation cohort (Nanchang)

Variable	RA (*n* = 236)	SjD (*n* = 83)	*P* value
Age	53.61 ± 13.78	52.53 ± 11.37	0.481
ESR (mm/h)	23.09 ± 17.91	18.17 ± 9.90	0.002
CRP (mg/L)	11.86 ± 25.10	2.21 ± 3.37	<0.001
RF (IU/mL)	107.52 ± 108.87	116.33 ± 233.68	0.741
TC (mmol/L)	4.55 ± 1.25	4.77 ± 1.82	0.302
TG (mmol/L)	1.44 ± 1.12	1.53 ± 0.83	0.478
HDL-C (mmol/L)	1.65 ± 0.57	1.53 ± 0.37	0.037
LDL-C (mmol/L)	2.64 ± 0.88	2.41 ± 0.69	0.015
Glucose (mmol/L)	5.57 ± 2.04	5.23 ± 0.70	0.027
ALT (U/L)	19.64 ± 12.43	19.55 ± 16.62	0.963
AST (U/L)	22.30 ± 11.02	23.77 ± 8.69	0.221
GGT (U/L)	28.02 ± 48.80	21.42 ± 16.99	0.074
Gender (male), *n* (%)	34 (14.4%)	2 (2.4%)	0.002
Hypertension, *n* (%)	12 (5.1%)	3 (3.6%)	0.767
Diabetes, *n* (%)	5 (2.1%)	0 (0.0%)	0.332
HCQ use, *n* (%)	57 (24.2%)	35 (42.2%)	0.006
GC use, *n* (%)	57 (24.2%)	67 (80.7%)	<0.001

Continuous variables are presented as mean ± SD; categorical variables as *n* (%). Independent samples *t* test was used for continuous variables; chi-square or Fisher’s exact test for categorical variables. The parametric test was selected for the external cohort given its smaller sample size and approximately normal distributions of key variables, whereas the non-parametric Wilcoxon rank-sum test was used for the larger discovery cohort (Table 1) to account for potential non-normality in skewed variables. Missing values (RF 17.6%, metabolic parameters 12.2–14.1%, liver enzymes 4.4–5.3%) were handled using multiple imputation by chained equations (MICE, *m* = 5, predictive mean matching, 20 iterations; seed = 2026). Complete case sensitivity analysis (*n* = 225) yielded AUC = 0.805 (95% CI: 0.739–0.871).

**Figure 6:**
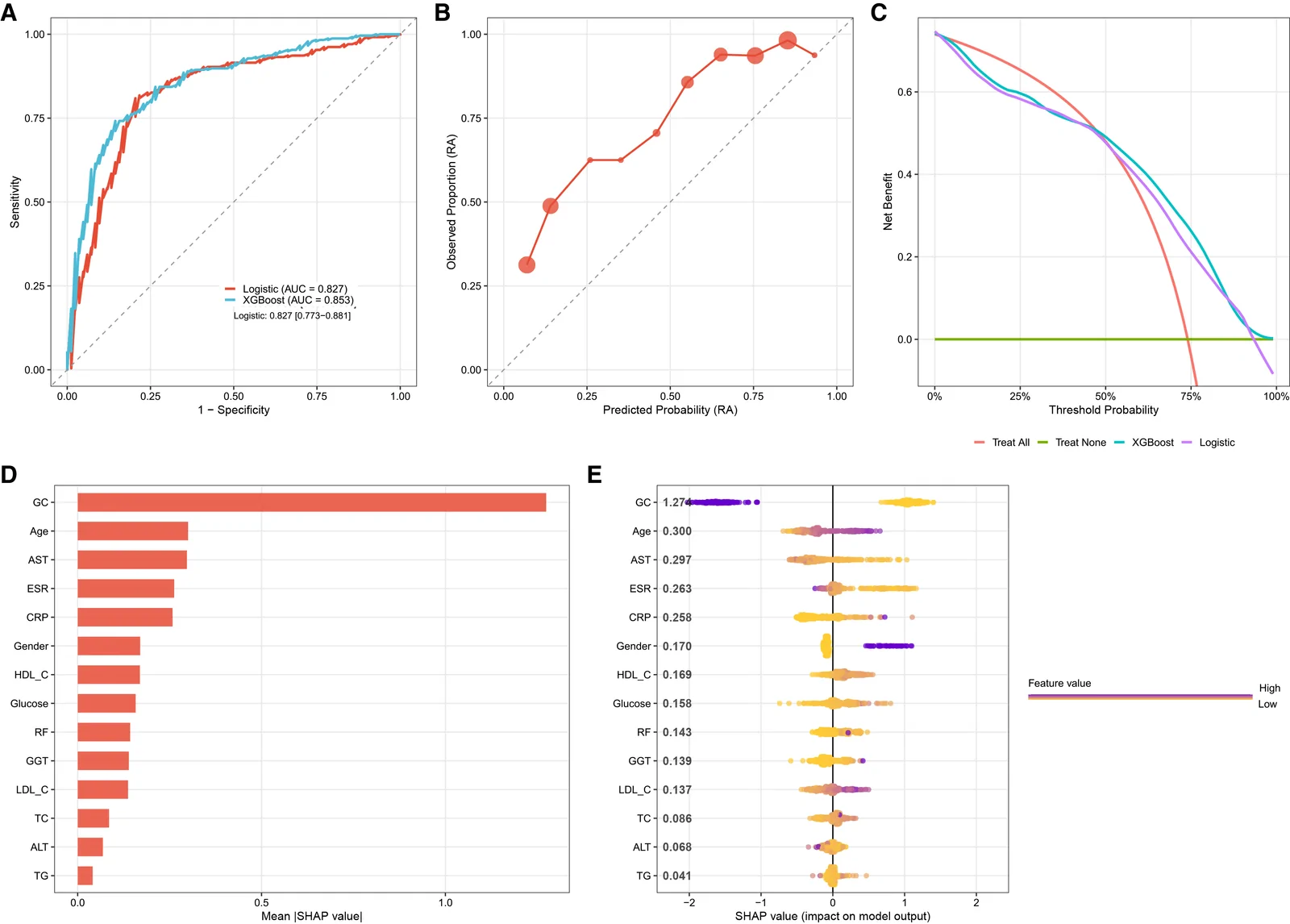
External validation in the Nanchang cohort (*n* = 319) (A) Receiver operating characteristic curve for the XGBoost model in the external validation cohort (pooled AUC = 0.840, 95% CI: 0.791–0.890). (B) Calibration plot shows agreement between predicted and observed probabilities. (C) Decision curve analysis demonstrates net clinical benefit across threshold probabilities. (D) SHAP feature importance in the external cohort. (E) SHAP summary plot in the external cohort.

The XGBoost model achieved a pooled AUC of 0.840 (95% CI: 0.791–0.890; Rubin’s rules across 5 MICE imputations) in the external validation cohort (Figure 6A), with the fraction of missing information being negligible (λ = 0.035), indicating that imputation uncertainty had minimal impact on AUC estimation. At the classification threshold of 0.465 (determined using Youden’s J statistic on the discovery cohort’s held-out test set and applied unchanged to the external cohort without re-optimization), the model achieved sensitivity of 0.750, specificity of 0.807, PPV of 0.917, and NPV of 0.532. Logistic regression achieved a comparable pooled AUC of 0.826 (95% CI: 0.772–0.880), consistent with the discovery cohort finding that both algorithms capture the underlying metabolic differences effectively. The calibration plot (Figure 6B) demonstrated reasonable agreement between predicted probabilities and observed frequencies in the external cohort. Quantitative calibration assessment (pooled across 5 MICE imputations) revealed a Brier score of 0.195, calibration intercept of 1.409, and calibration slope of 1.225 for the XGBoost model, indicating that while discrimination is preserved, predicted probabilities require recalibration for accurate risk estimation in this population (Table S4). The logistic regression model showed comparable external calibration (pooled Brier = 0.207), suggesting that simpler models may generalize more stably across populations. DCA (Figure 6C) confirmed that the model retained net clinical benefit across a broad threshold range in the independent population.

Subgroup analysis of the external validation cohort (Table 5) showed consistent performance across clinically relevant strata: AUC of 0.846 (95% CI: 0.791–0.900) in patients aged ≤60 years, 0.826 (0.718–0.933) in those aged >60 years, and 0.839 (0.788–0.890) in females. Performance was maintained in HCQ users (AUC = 0.874) and non-users (AUC = 0.840), further confirming that model discrimination is not dependent on HCQ-related metabolic confounding. The male subgroup had insufficient SjD sample size for reliable analysis (*n* = 2). Among patients with high RF titers (>200 IU/mL), the model achieved an AUC of 0.880 (95% CI: 0.753–1.000), though with a small SjD sample (*n* = 10).

**Table 5:** Subgroup analysis of external validation cohort

Subgroup	N (RA/SjD)	AUC (95% CI)
Age ≤ 60	230 (161/69)	0.843 (0.787–0.898)
Age > 60	89 (75/14)	0.840 (0.713–0.967)
Female	283 (202/81)	0.852 (0.803–0.901)
Male	36 (34/2)	NA^a^
RF ≤ 200 IU/mL	269 (196/73)	0.842 (0.790–0.894)
RF > 200 IU/mL	50 (40/10)	0.875 (0.748–1.000)^b^
HCQ user	92 (57/35)	0.861 (0.784–0.938)
HCQ non-user	225 (177/48)	0.833 (0.766–0.899)
GC user	124 (57/67)	0.673 (0.578–0.768)
GC non-user	195 (179/16)	0.696 (0.567–0.826)

a Insufficient SjD sample size for reliable subgroup analysis (*n* = 2 for male subgroup).

b Small SjD sample size (*n* = 10); results should be interpreted with caution.

SHAP analysis of the external validation cohort (Figures 6D and 6E) revealed a feature importance ranking broadly consistent with the discovery cohort: GC remained the dominant feature (mean |SHAP| = 1.274), followed by Age (0.300), AST (0.297), ESR (0.263), and CRP (0.258). The preservation of GC, AST, and CRP among the top-ranking features across two independent populations strongly supports the biological reproducibility of the identified metabolic signatures.

### Transcriptomic corroboration of metabolic pathway divergence

**Figure 7:**
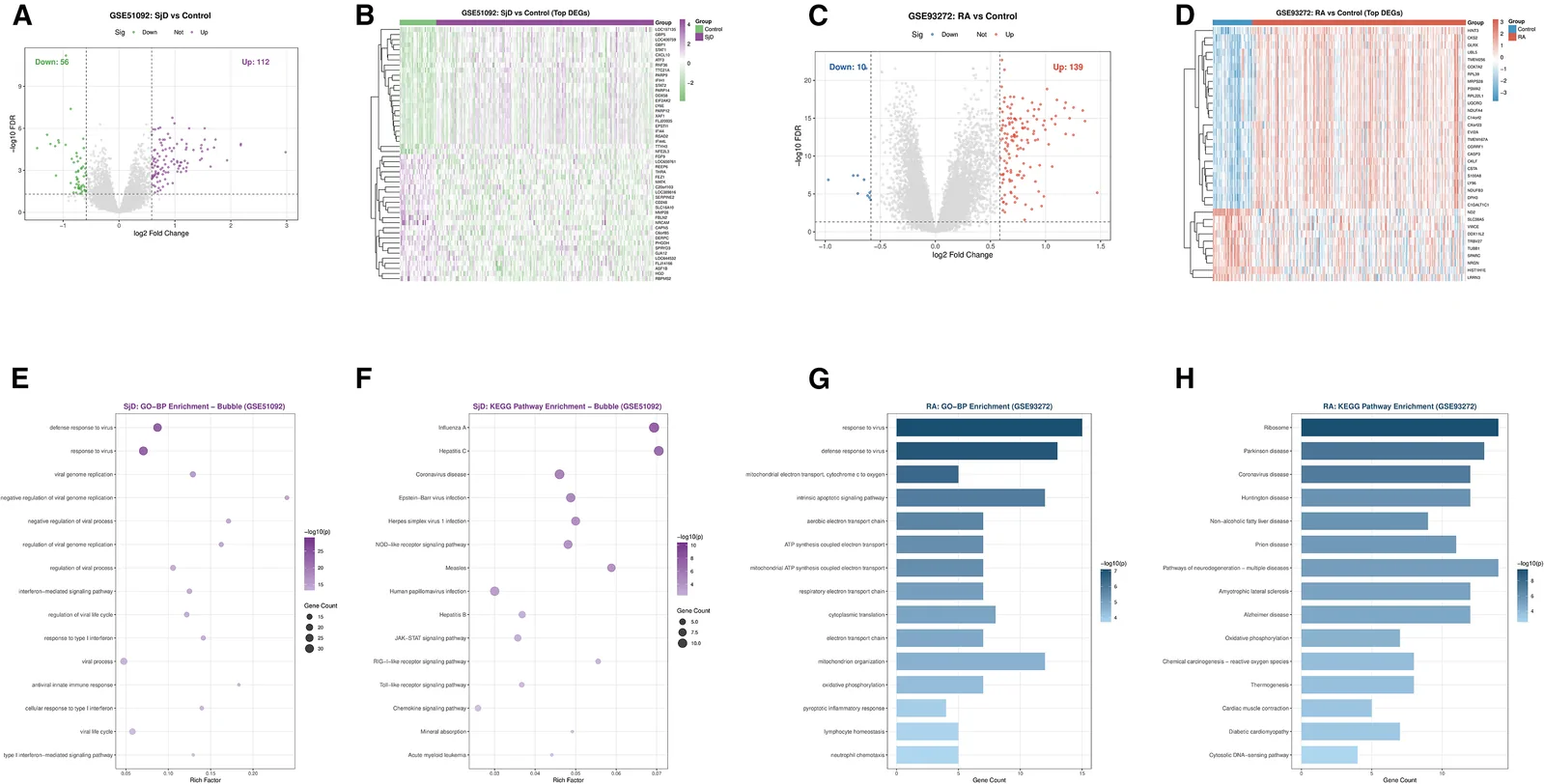
Transcriptomic corroboration of metabolic pathway divergence between pSS and RA Parallel differential expression analyses of publicly available PBMC datasets (GSE51092 for pSS, GSE93272 for RA). (A) pSS volcano plot. The most prominently upregulated genes were type I interferon-stimulated genes: ISG15, IFI6, MX1, OAS1, OAS2, OAS3, RSAD2, IFI44L, IFI44, IFIT1, IFIT3, and HERC5. (B) pSS heatmap of the top 20 DEGs showing consistent ISG upregulation across patient samples. (C) RA volcano plot shows the upregulation of mitochondrial genes (UQCRQ, COX7A2, NDUFA4, COX6C), ribosomal protein genes (RPS7, RPL39), ISGs shared with pSS (IFI6, IFIT1, IFI44, CXCL10, ISG15, RSAD2, IFI44L, HERC5), and inflammatory mediators (S100A8, TNFAIP6). (D) RA heatmap of top 20 DEGs. (E) pSS GO-BP enrichment dominated by type I interferon signaling and antiviral defense terms. (F) pSS KEGG enrichment shows JAK-STAT signaling and viral infection-related pathways. (G) RA GO-BP enrichment dominated by mitochondrial electron transport, oxidative phosphorylation, and aerobic respiration terms. (H) RA KEGG enrichment dominated by ribosome and oxidative phosphorylation pathways. Significance thresholds: |log_2_FC| > 1 and adjusted *p* < 0.05 (Benjamini-Hochberg).

To explore the molecular basis underlying the clinically observed metabolic differences between RA and SjD, we performed parallel transcriptomic analyses using two publicly available PBMC datasets: GSE51092 (pSS, *n* = 190 vs. 32 healthy controls) and GSE93272 (RA, *n* = 232 vs. 43 healthy controls). Each disease was compared independently against its own matched controls to avoid cross-platform batch effects. Differential expression analysis revealed fundamentally distinct transcriptional programs (Figures 7A–7D). The pSS transcriptome was dominated by canonical type I interferon-stimulated genes (ISGs), including ISG15, IFI6, MX1, and OAS1–3, with GO-BP enrichment overwhelmingly concentrated in type I interferon signaling and antiviral defense pathways (Figure 7E). KEGG analysis confirmed enrichment for JAK-STAT signaling and viral infection-related pathways (Figure 7F). In contrast, the RA transcriptome was characterized by upregulation of mitochondrial respiratory chain genes (UQCRQ, COX7A2, NDUFA4) and ribosomal protein genes, with GO-BP enrichment dominated by mitochondrial electron transport, oxidative phosphorylation, and aerobic respiration (Figure 7G). KEGG analysis showed ribosome and oxidative phosphorylation as the top enriched pathways (Figure 7H). Notably, 13 ISGs were shared between both diseases, indicating a common but subordinate interferon activation module alongside their divergent dominant programs. These transcriptomic findings provide molecular-level corroborative evidence consistent with the clinically observed metabolic divergence. The dominant mitochondrial and oxidative phosphorylation gene signature in RA PBMCs likely reflects the broader metabolic reprogramming occurring within heterogeneous immune cell populations: while pro-inflammatory effector cells (M1 macrophages, Th17 cells) shift toward aerobic glycolysis, other PBMC subsets may compensatory upregulate mitochondrial gene programs, and increased mitochondrial biogenesis itself is a recognized hallmark of activated immune cells adapting to heightened energy demands. This transcriptional landscape is thus compatible with the hyperglycemic profile observed clinically, as the overall metabolic milieu reflects increased glucose consumption by glycolytic immune effectors alongside mitochondrial adaptations in other cell populations. Conversely, the IFN-I-dominant transcriptional architecture in pSS corroborates the observation that SjD exhibits lower CRP despite comparable ESR, as type I interferon is a comparatively weak inducer of hepatic CRP synthesis. Together, these parallel but independent analyses reinforce the biological plausibility of using routine metabolic biomarkers to distinguish these two diseases. It should be noted that the transcriptomic datasets represent general pSS and RA populations rather than the specific high-inflammatory SjD phenotype studied clinically; the pathway-level divergence nonetheless provides a biological framework for understanding why metabolic differences exist between these two disease categories.

We emphasize that the transcriptomic analyses are observational, based on different patient populations (general pSS and RA) from publicly available datasets; they provide pathway-level biological plausibility but do not establish causal relationships between transcriptomic programs and the metabolic biomarkers used in our model.

## Discussion

This study demonstrates that a clinical-metabolic combined model integrating demographic characteristics (sex, age), inflammatory status (CRP), and metabolic biomarkers (glucose, liver enzymes) can provide meaningful auxiliary discriminating information to differentiate RA from the high-inflammatory articular phenotype of SjD. Critically, external validation in an independent Nanchang cohort (pooled AUC = 0.840) confirmed the generalizability of this approach across geographically and clinically distinct populations. RA is characterized by elevated glucose, higher CRP, and atherogenic lipid elevation, while SjD-Phenotype 1 presents with elevated liver transaminases. These findings are particularly valuable for patients in the clinical gray zone where anti-CCP is negative or indeterminate.

The persistent significance of elevated glucose as an independent RA discriminator after dual adjustment for both GC and HCQ use (OR = 1.20, *p* = 0.028; Table S1) strongly implicates intrinsic metabolic dysregulation rather than medication-driven artifact. This finding aligns with accumulating evidence that RA represents not merely a joint disease but a systemic metabolic disorder.^13^^,^^14^ At the molecular level, chronic exposure to TNF-α and IL-6—hallmark cytokines of RA synovitis—impairs insulin signaling by promoting inhibitory serine phosphorylation of insulin receptor substrate-1 (IRS-1), thereby inducing systemic insulin resistance independently of GC exposure.^22^^,^^23^ Furthermore, activated immune cells central to RA pathogenesis—particularly M1-polarized macrophages and Th17 lymphocytes—undergo metabolic reprogramming toward aerobic glycolysis (the Warburg effect), competing with peripheral tissues for circulating glucose and contributing to the hyperglycemic milieu.^17^^,^^24^ Critically, this metabolic signature appears distinct from SjD, where the predominant pathogenic mechanism—B-cell hyperactivation and lymphocytic glandular infiltration—engages different metabolic programs centered on oxidative phosphorylation and fatty acid oxidation.^16^ Our transcriptomic analysis provides molecular-level corroborative evidence for this divergence: RA PBMCs exhibited dominant upregulation of mitochondrial respiratory chain genes (UQCRQ, COX7A2, NDUFA4) with pathway enrichment in oxidative phosphorylation, whereas pSS PBMCs were characterized by type I ISG programs (Figure 7). The divergent metabolic demands of these fundamentally different immune effector pathways may thus explain the differential glucose profiles observed clinically, providing a mechanistic rationale for glucose as a disease-discriminating biomarker.

The observation that SjD-Phenotype 1 patients exhibited an AST-predominant transaminase pattern (AST 25.3 vs. ALT 23.5 U/L) warrants particular attention, as it suggests a tissue origin distinct from hepatocellular injury. Unlike ALT, which is predominantly hepatic, AST is abundantly expressed in skeletal muscle, cardiac muscle, and salivary glands. The AST > ALT pattern observed in SjD is more consistent with myogenic rather than hepatogenic injury,^25^ raising the hypothesis that subclinical muscle involvement may be underrecognized in SjD. Indeed, myositis in SjD has been reported with a prevalence of up to 3–9% when systematically assessed, and subtle elevations in muscle-derived enzymes may indicate subclinical inflammatory myopathy below the threshold of clinical detection.^25^^,^^26^ Additionally, lymphocytic infiltration of salivary glands—the pathological hallmark of SjD—may contribute to circulating AST through local tissue damage, as salivary acinar cells express mitochondrial AST. The non-linear threshold effect of AST revealed by SHAP analysis (Figure 3C) is particularly illuminating: AST contributes to SjD classification predominantly when markedly elevated, a pattern consistent with tissue injury reaching a threshold of clinical significance rather than a gradual linear relationship. This mechanistic interpretation supports the finding that linear regression models, which assume monotonic relationships, fail to capture this biologically meaningful threshold effect. Future studies incorporating creatine kinase (CK) and aldolase measurements would help clarify the relative contributions of hepatic versus myogenic AST elevation in SjD and potentially refine the diagnostic model.

The strong discriminative value of CRP for RA (OR = 2.13) despite both diseases sharing high-inflammatory features merits mechanistic explanation. Importantly, RA and SjD-Phenotype 1 both exhibited elevated ESR (49.5 vs. 51.2 mm/h, *p* = 0.112), yet CRP levels diverged significantly (25.2 vs. 15.3 mg/L, *p* < 0.001). This dissociation between ESR and CRP reflects fundamentally different inflammatory architectures. CRP synthesis in hepatocytes is predominantly driven by IL-6 via the JAK-STAT3 signaling cascade, and RA is characterized by robust IL-6 production from inflamed synovium, making CRP a sensitive surrogate for synovitis-driven systemic inflammation.^27^ In contrast, the inflammatory signature of SjD is dominated by type I interferon (IFN-I) pathway activation and polyclonal B-cell hyperactivation, which are comparatively weak inducers of hepatic CRP synthesis.^18^^,^^27^ Meanwhile, ESR elevation in SjD is partly explained by hypergammaglobulinemia—a hallmark of B-cell-driven disease—which increases erythrocyte aggregation independently of acute-phase protein production. Thus, the CRP-ESR dissociation observed between RA and SjD-Phenotype 1 is not paradoxical but rather a direct consequence of IL-6-dominant (RA) versus IFN-I/B-cell-dominant (SjD) inflammatory pathways, each producing distinct biochemical footprints in routine laboratory tests. This CRP-ESR dissociation pattern was also evident in the external validation cohort: although ESR showed a modest statistical difference (23.1 vs. 18.2 mm/h, *p* = 0.002), the absolute difference was clinically trivial (∼5 mm/h), whereas CRP differed by approximately 5-fold (11.9 vs. 2.2 mg/L, *p* < 0.001). Our transcriptomic analysis reinforced this interpretation: pSS PBMCs showed overwhelmingly dominant interferon pathway enrichment (Figures 7E and 7F), while RA PBMCs exhibited mitochondrial and metabolic pathway dominance (Figures 7G and 7H), with shared but subordinate ISG modules in both diseases.

A particularly noteworthy aspect of our HCQ confounding analyses is that the direction of confounding actually strengthens rather than weakens our findings. HCQ has well-documented glucose-lowering effects through improved insulin sensitivity and reduced hepatic gluconeogenesis,^28^^,^^29^ and was used at significantly higher rates in the SjD-Phenotype 1 group (43.4% vs. 3.4%). This differential HCQ exposure would be expected to lower glucose levels preferentially in the SjD group, thereby artificially narrowing the true inter-group glucose difference. Consistent with this reasoning, dual adjustment for both GC and HCQ resulted in only modest attenuation of the glucose odds ratio (from 1.30 to 1.20), and the HCQ-stratified analysis demonstrated that among HCQ non-users—a population entirely free from this confounding influence—the glucose difference persisted with high significance (5.38 vs. 4.93 mmol/L, *p* < 0.001). This pattern of “reverse confounding,” wherein the confounder leads to underestimation of the true effect, provides an unusually robust form of methodological reassurance. It implies that the true metabolic divergence between RA and SjD may be even greater than what our model captures, and that our reported AUC values represent conservative estimates of the model’s discriminative potential in settings where HCQ exposure is either controlled for or absent.

The atherogenic lipid profile observed in RA (elevated TC, HDL-C, and LDL-C) reflects the interplay between chronic systemic inflammation and lipid metabolism. While elevated HDL-C is traditionally considered cardioprotective, accumulating evidence demonstrates that HDL particles in RA undergo qualitative transformation into “pro-inflammatory HDL” with impaired cholesterol efflux capacity and reduced antioxidant function, despite quantitatively elevated levels.^30^^,^^31^ The IL-6-driven hepatic acute-phase response in RA simultaneously increases production of serum amyloid A (SAA), which displaces apolipoprotein A-I from HDL particles, fundamentally altering their function. In contrast, the SjD inflammatory milieu, dominated by IFN-I and lymphocytic glandular infiltration rather than systemic IL-6-driven inflammation, may produce less pronounced perturbation of hepatic lipid metabolism. The observation that lipid differences (TC, HDL-C, LDL-C) persisted as independent discriminators in multivariable analysis supports the concept that RA and SjD produce qualitatively different systemic metabolic consequences despite sharing superficial serological similarities such as RF positivity.

Male sex was the strongest predictor for RA classification (OR = 4.17), reflecting the epidemiological difference between the two diseases. Critically, sex-stratified analysis showed an AUC of 0.902 in the female subgroup of the discovery cohort and 0.852 in the external validation cohort, demonstrating that metabolic features retain strong independent discriminative ability even after excluding sex as a factor. RF showed no discriminative value in this study, which is an expected result of study design, as SjD-Phenotype 1 is defined by high RF titers—this is precisely the starting point for our search for alternative markers.

The external validation results merit particular discussion. The Nanchang cohort differed substantially from the Tianjin discovery cohort in several respects: patients were younger (mean 53.6 vs. 62.9 years), had lower overall inflammation (mean CRP 11.9 mg/L in the external cohort vs. 25.2 mg/L in the discovery cohort), and showed different treatment patterns (GC use 24.2% vs. 13.7% in RA). Despite these inter-center differences, the model achieved a pooled AUC of 0.840, with consistent SHAP feature importance rankings (GC, AST, and CRP remaining among the top features in both cohorts). This cross-center generalizability is particularly encouraging given that the features used in our model are internationally standardized laboratory tests, facilitating reproducibility across healthcare systems. The slightly lower AUC in the external cohort compared to internal validation (0.840 vs. 0.88) is expected and reflects the natural performance decrement observed when models are applied to populations with different demographic and clinical characteristics—a phenomenon that paradoxically strengthens confidence in the model’s generalizability rather than undermining it.

Notably, glucose—a core metabolic discriminator in the discovery cohort (OR = 1.30, *p* < 0.001)—showed a modest but statistically significant difference in the external validation cohort (5.57 ± 2.04 vs. 5.23 ± 0.70 mmol/L, *p* = 0.027, Table 4), though the absolute difference was small (0.34 mmol/L). The persistence of glucose significance across both cohorts, despite different population characteristics and treatment patterns, reinforces the biological plausibility of glucose as an intrinsic RA metabolic discriminator. However, TC and HDL-C—both independent discriminators in the discovery cohort—did not reach significance in the external validation cohort after accounting for multiple comparisons (*p* = 0.302 and *p* = 0.037, respectively), illustrating the principle of multi-feature complementarity: the relative contribution of individual features can shift across populations while the ensemble maintains discriminative power through feature complementarity. In the external cohort, the substantially larger CRP difference (11.9 vs. 2.2 mg/L, *p* < 0.001) and stronger GC use disparity (24.2% vs. 80.7%) ensured robust overall model performance (pooled AUC = 0.840), strengthening the clinical argument for multi-feature models over single-biomarker approaches.

Our validation analysis in the ACPA-negative RA subgroup showed highly consistent performance with the main analysis (AUC = 0.910 vs. 0.911), directly confirming the model’s applicability to the clinical gray zone where anti-CCP is negative or indeterminate. Clinical differentiation between RA and SjD remains challenging, with misdiagnosis of SjD as RA being not uncommon,^32^ and some SjD patients may even be anti-CCP positive,^33^^,^^34^ creating diagnostic dilemmas when relying solely on serology.

A key strength is the integration of SHAP analysis to achieve model interpretability. SHAP dependence plots revealed non-linear relationships that logistic regression cannot capture—for example, AST’s discriminative effect is most pronounced at elevated levels, suggesting a threshold effect consistent with subclinical tissue involvement. Importantly, this SHAP feature ranking was replicated in the external validation cohort (Figure 6D), demonstrating that the identified non-linear patterns are reproducible rather than cohort-specific artifacts. These insights may guide future optimization of clinical decision rules.

Regarding clinical impact, our findings translate into a practical three-step auxiliary diagnostic framework for the challenging scenario of polyarthritis with RF positivity and anti-CCP negativity or indeterminate results. Step 1 (prior probability): sex information provides an important initial estimate—male patients are substantially more likely to have RA (OR = 4.17), given the extreme female predominance of SjD (female:male approximately 9:1). Step 2 (metabolic assessment): among routine laboratory tests already available at the time of initial evaluation, elevated fasting glucose (OR = 1.30), higher CRP (OR = 2.13), and an atherogenic lipid profile (elevated TC, HDL-C, LDL-C) favor RA, while elevated AST—particularly when markedly elevated (SHAP threshold effect)—and concurrent GC therapy favor SjD. Step 3 (integrated decision): these metabolic clues should be integrated with, not replace, traditional clinical assessment including anti-Ro/SSA testing, salivary gland evaluation, and imaging. The key clinical advantage of this approach is its immediate accessibility: glucose, CRP, lipid profiles, and liver function tests are universally available, internationally standardized, and already part of routine care—no additional testing is required, only a new interpretive framework for existing data. Future development of a simplified scoring tool or web-based calculator incorporating these features would further facilitate bedside implementation.

### Limitations of the study

This study has several limitations. First, although external validation in an independent cohort from the First Affiliated Hospital of Nanchang University confirmed model generalizability across geographically distinct populations within China, the external validation was performed at a single center; multi-center external validation across additional domestic and international sites, encompassing different genetic backgrounds, dietary habits, and healthcare systems, is needed for broader applicability. Second, the cross-sectional design cannot assess diagnostic utility in early disease. Third, we did not include anti-CCP, anti-Ro/SSA, and anti-La/SSB antibodies as model features because this model is explicitly positioned for the clinical gray zone where anti-CCP is negative or indeterminate, with ACPA-negative validation (AUC = 0.910) confirming this approach. Fourth, although we excluded clearly diagnosed liver diseases, undiagnosed mild hepatic lesions may exist, and CK data were unavailable to distinguish hepatogenic from myogenic AST elevation. Fifth, although our HCQ confounding analyses consistently demonstrated that key metabolic differences persist after accounting for HCQ, HCQ dosage and duration data were unavailable, precluding dose-response analysis. Sixth, the transcriptomic analysis used publicly available datasets from different study designs and platforms; while the parallel analysis strategy (each disease vs. its own matched controls) avoids batch effects, direct cross-disease transcriptomic comparison was not performed. Importantly, GC use was the strongest discriminating feature in both SHAP and logistic regression analyses, raising the question of whether the model captures treatment patterns rather than intrinsic biological differences; however, metabolic features (glucose, CRP, lipids) retained independent significance after GC adjustment, and the GC non-user subgroup still achieved a positive AUC, supporting the presence of genuine biological signal beyond treatment background. Nonetheless, the GC non-user subgroup in the external validation cohort showed a lower AUC of 0.696 (95% CI: 0.567–0.826) with only 16 SjD patients, indicating that model performance may be attenuated in populations where GC use—the strongest discriminating feature—is absent; larger GC non-user cohorts are needed to confirm performance in this subgroup. The relatively low NPV (0.532) in external validation suggests that approximately 47% of patients predicted as SjD were actually RA, underscoring that this model should serve as an auxiliary tool integrated with clinical assessment rather than a standalone diagnostic instrument. Furthermore, the external validation cohort had substantial missing data in laboratory variables (up to 17.6% for RF), necessitating MICE multiple imputation; although complete case sensitivity analysis (AUC = 0.805, *n* = 225) supported the robustness of findings, the impact of imputation on individual feature contributions cannot be fully excluded. Additionally, RF levels in the external validation SjD subgroup (mean 116.33 IU/mL) were lower than in the discovery cohort (mean 216.74 IU/mL), and the inter-group RF relationship differed (SjD > RA in the discovery cohort vs. comparable levels in external validation, *p* = 0.741), likely reflecting the difference between data-driven phenotype identification through unsupervised clustering and criteria-based manual classification in the external cohort. The GC user subgroup in the external validation cohort showed an AUC of 0.673, below the conventional 0.7 threshold, indicating attenuated performance when GC use is prevalent in both groups and the model’s strongest discriminating feature contributes less to classification. Finally, the small number of SjD-Phenotype 1 patients with diabetes limits analysis of this subgroup. Multi-center international validation studies, particularly those incorporating diverse ethnic populations, remain a priority for future research. Seventh, model ablation analysis revealed that GC is the strongest single discriminating feature, and the treatment-only model (pooled AUC = 0.840) outperformed the metabolic-only model (AUC = 0.716). While metabolic features provide significant incremental value (ΔAUC = +0.086, *p* < 0.001) and retain discriminative ability in GC-matched and GC-stratified populations, model performance is substantially enhanced by treatment pattern differences between groups. Future studies should evaluate whether the metabolic-only model alone performs adequately in settings where treatment histories are unavailable. Eighth, the XGBoost model showed suboptimal calibration in external validation (calibration intercept = 1.409, calibration slope = 1.225, Brier score = 0.195), indicating that while discrimination is preserved (pooled AUC = 0.840), predicted probabilities still require recalibration before clinical deployment in new populations. Recalibration strategies such as Platt scaling should be evaluated in future work. Ninth, the ACPA-negative subgroup analysis (AUC = 0.910) was performed on data that included training samples and should be considered exploratory; fully held-out validation in ACPA-negative cohorts is needed.

In summary, this study constructed a clinical-metabolic combined model that can provide auxiliary information for differentiating RA from the high-inflammatory articular phenotype of SjD. External validation in an independent cohort confirmed the model’s generalizability, and transcriptomic pathway analysis provided molecular-level corroboration for the observed metabolic divergence. In the clinical gray zone where anti-CCP is negative or indeterminate, routine biochemical parameters—particularly glucose, CRP, and liver transaminases—provide discriminating information independent of and complementary to traditional autoantibodies.

## Resource availability

### Lead contact

Further information and requests for resources should be directed to and will be fulfilled by the lead contact, Wei Liu (fengshiliuwei@163.com).

### Materials availability

This study did not generate new unique reagents.

### Data and code availability

• The clinical data reported in this study are available from the lead contact upon reasonable request, subject to approval by the institutional ethics committee, as they contain sensitive patient information.
• All R code for statistical analysis, model training, external validation, ablation analysis, calibration assessment, and transcriptomic analysis has been deposited on GitHub (https://github.com/a1610264527/RA-SjD-Metabolic-Model) and archived with a persistent DOI at Zenodo: https://doi.org/10.5281/zenodo.21634114. The repository includes complete model specifications (hyperparameters, feature list, preprocessing pipeline, and random seeds) to enable full reproducibility.
• The publicly available transcriptomic datasets used in this study are accessible from the Gene Expression Omnibus (GEO) repository under accession numbers GEO: GSE51092 and GEO: GSE93272.
• Any additional information required to reanalyze the data reported in this paper is available from the lead contact upon request.

## Acknowledgments

The researchers sincerely thank all the participants and healthcare professionals involved in this study for their support and cooperation. We thank the Department of Rheumatology and Immunology, the First Affiliated Hospital of Nanchang University, for providing external validation data.

This study was supported by the following projects: Major Difficult Disease Integrated Traditional Chinese and Western Medicine Clinical Collaboration Project—Gout and Hyperuricemia (project no. ZDYN-2024-B-038); Tianjin Nankai District Traditional Chinese Medicine Heritage and Innovation Development Demonstration Pilot Project—Syndrome Pattern Research and Expert Consensus Formulation in Rheumatic Diseases (Rheumatoid Arthritis, Sjögren disease, and Gout) (project no. 20240204011); National Administration of Traditional Chinese Medicine Project—Research on Integrated Traditional Chinese and Western Medicine Diagnosis and Treatment Protocols for Major Difficult Diseases (Integrated Traditional Chinese and Western Medicine Diagnosis and Treatment Guidelines for Sjögren disease) (project no. 2023382); National Famous Traditional Chinese Medicine Expert Inheritance Studio (project no. 975022); Study on the Efficacy and Mechanism of Yin-Nourishing and Detoxifying Traditional Chinese Medicine in the Treatment of Sjögren disease (project no. GZY-KJS-2024-05); Tianjin Municipal Health Commission Traditional Chinese and Western Medicine Integrated Research Institute Construction Project—Tianjin Integrated Traditional Chinese and Western Medicine Rheumatic Disease Research Institute; 10.13039/501100012151Sanming Project of Medicine in Shenzhen (no. SZZYSM202403014), and the Major Science and Technology Project of the Health System of Nanshan District, Shenzhen (no. NSZD2025013).

## Author contributions

J.B.L., S.R.L., and R.H.L. contributed to conceptualization, data curation, formal analysis, investigation, methodology, software, writing – original draft, and writing – review and editing. Y.Z.G., N.T., and X.Q.W. contributed to data curation, formal analysis, investigation, methodology, and resources. M.X.L. contributed to external validation data collection, curation, and resources. W.L. contributed to conceptualization, data curation, formal analysis, investigation, methodology, resources, software, supervision, validation, visualization, writing – original draft, and writing – review and editing.

## Declaration of interests

The authors declare no competing interests.

## Declaration of generative AI and AI-assisted technologies in the writing process

During the preparation of this manuscript, the authors used AI-assisted tools (Claude, Anthropic) to improve language clarity and readability of the English text. After using these tools, the authors carefully reviewed and edited all content, and take full responsibility for the accuracy and integrity of the published work. All data analysis, interpretation of results, and scientific conclusions were performed independently by the authors without AI assistance.

## STAR★Methods

### Key resources table

**Table:** 

REAGENT or RESOURCE	SOURCE	IDENTIFIER
**Deposited data**		

Clinical data from First Teaching Hospital of Tianjin University of TCM (2014–2024) — Discovery cohort	Hospital clinical database	N/A
External validation data from the First Affiliated Hospital of Nanchang University (2022–2023)	Hospital clinical database	N/A
GSE51092 (pSS PBMC transcriptome)	NCBI GEO	GEO: GSE51092
GSE93272 (RA PBMC transcriptome)	NCBI GEO	GEO: GSE93272

**Software and algorithms**		

R version 4.5.0	R Software Foundation	https://cran.r-project.org/
xgboost (v3.1.3.1)	CRAN	https://cran.r-project.org/package=xgboost
SHAPforxgboost (v0.2.0)	CRAN	https://cran.r-project.org/package=SHAPforxgboost
pROC (v1.19.0.1)	CRAN	https://cran.r-project.org/package=pROC
caret (v7.0-1)	CRAN	https://cran.r-project.org/package=caret
ggplot2 (v4.0.1)	CRAN	https://cran.r-project.org/package=ggplot2
dcurves (v0.5.1)	CRAN	https://cran.r-project.org/package=dcurves
limma	Bioconductor	https://bioconductor.org/packages/limma
clusterProfiler	Bioconductor	https://bioconductor.org/packages/clusterProfiler
GEOquery	Bioconductor	https://bioconductor.org/packages/GEOquery
pheatmap	CRAN	https://cran.r-project.org/package=pheatmap
mice (v3.16.0)	CRAN	https://cran.r-project.org/package=mice
Analysis code and model specifications	This paper	https://github.com/a1610264527/RA-SjD-Metabolic-Model; https://doi.org/10.5281/zenodo.21634114

### Experimental model and study participant details

#### Study design and participants

This was a multi-center retrospective study with a discovery cohort and an external validation cohort.

##### Discovery cohort (Tianjin)

Data were collected from the clinical database of the First Teaching Hospital of Tianjin University of Traditional Chinese Medicine from January 2014 to December 2024. RA patients (*n* = 4,736) all met the 2010 ACR/EULAR classification criteria.^35^ The SjD cohort was derived from our previously published cluster analysis study, which used unsupervised machine learning to identify distinct disease phenotypes.^7^ This study specifically selected patients classified as Phenotype 1 (*n* = 594), characterized by high RF titers, elevated inflammatory markers, and prevalent joint involvement, representing the subgroup most clinically similar to RA.

##### External validation cohort (Nanchang)

An independent external validation cohort was recruited from the Department of Rheumatology and Immunology, the First Affiliated Hospital of Nanchang University (Nanchang, Jiangxi Province, China), from January 2022 to January 2023, comprising 236 RA and 83 SjD-Phenotype 1 patients. RA patients met the 2010 ACR/EULAR classification criteria,^35^ and SjD-Phenotype 1 patients were classified using the same clinical criteria derived from the discovery cohort’s cluster analysis,^7^ specifically requiring RF positivity with elevated inflammatory markers (ESR or CRP above the upper limit of normal) and documented articular involvement, ensuring phenotypic consistency across cohorts. Specifically, SjD-Phenotype 1 patients in the external cohort were classified using operationalized criteria: (1) fulfillment of 2016 ACR/EULAR classification criteria for primary Sjögren’s syndrome; (2) RF positivity (>20 IU/mL); (3) at least one elevated inflammatory marker (ESR or CRP above laboratory upper limit of normal); and (4) documented articular involvement. Classification was performed independently by two rheumatologists at the Nanchang center, with disagreements resolved by consensus. The same inclusion and exclusion criteria as the discovery cohort were applied. This cohort, geographically located approximately 1,200 km from the discovery center in Tianjin, served as an independent external validation set to assess model generalizability across different healthcare settings and patient populations.

Inclusion criteria required complete laboratory data for all metabolic and inflammatory variables of interest. Exclusion criteria included: (1) overlap syndromes or secondary Sjögren disease; (2) known viral hepatitis (hepatitis B surface antigen positive or hepatitis C antibody positive); (3) radiologically or clinically diagnosed non-alcoholic fatty liver disease; (4) primary biliary cholangitis (PBC) or autoimmune hepatitis (AIH); (5) history of chronic heavy alcohol consumption (>40g/day for men, >20g/day for women); (6) missing key variables (for the discovery cohort). These exclusion criteria were designed to ensure that observed liver enzyme differences reflect intrinsic disease biology rather than comorbid liver conditions.

All study participants were adult human patients (Homo sapiens) of Han Chinese ancestry. In the discovery cohort, the mean age at assessment was 62.85 ± 11.23 years for RA patients (*n* = 4,736; 78.5% female, 21.5% male) and 57.41 ± 12.36 years for SjD-Phenotype 1 patients (*n* = 594; 93.6% female, 6.4% male). In the external validation cohort, the mean age was 53.61 ± 13.78 years for RA patients (*n* = 236; 85.6% female, 14.4% male) and 52.53 ± 11.37 years for SjD-Phenotype 1 patients (*n* = 83; 97.6% female, 2.4% male). No restrictions on age range were applied; all patients meeting the classification criteria and not excluded by the above criteria were included. Detailed baseline characteristics including age, sex distribution, and comorbidities for both cohorts are presented in Tables 1 and 4.

#### Ethics approval

This study was approved by the Ethics Review Committee of the First Teaching Hospital of Tianjin University of Traditional Chinese Medicine (approval number: TYLL2026[K]027) and the Ethics Review Committee of the First Affiliated Hospital of Nanchang University (approval number: IIT[2023]Linlun No. 045). Since this study was a retrospective analysis, both ethics committees waived the requirement for obtaining patient informed consent.

Sex was included as a covariate in all multivariable analyses. Sex-stratified subgroup analysis demonstrated that the model maintained strong discriminative performance in the female subgroup (AUC = 0.902, 95% CI: 0.888–0.915), confirming that the model’s predictive ability is not solely driven by sex differences between the two diseases. A limitation of this study is that sex-specific biological mechanisms underlying the observed metabolic differences were not further explored and warrant investigation in future studies.

### Method details

#### Clinical and laboratory variables

Demographic variables included age at assessment and sex. Inflammatory markers included erythrocyte sedimentation rate (ESR), C-reactive protein (CRP), and rheumatoid factor (RF). Metabolic parameters included fasting glucose, total cholesterol (TC), triglycerides (TG), high-density lipoprotein cholesterol (HDL-C), and low-density lipoprotein cholesterol (LDL-C). Liver enzymes included alanine aminotransferase (ALT), aspartate aminotransferase (AST), and γ-glutamyl transferase (GGT). Comorbidities (hypertension, diabetes) and medication use (hydroxychloroquine, methotrexate, leflunomide, glucocorticoids) were also recorded. Glucocorticoid (GC) use was defined as a binary variable (yes/no) indicating whether the patient was receiving any oral glucocorticoid therapy at the time of laboratory assessment, regardless of dosage or duration. This pragmatic definition was adopted because: (1) in routine clinical practice, the decision to prescribe GC reflects the treating physician’s assessment of disease activity and severity, making GC use itself an informative clinical feature; (2) detailed dosage and duration data were not consistently available across all records in this retrospective database. GC was not “normalized” in the traditional sense but rather incorporated as a binary covariate in multivariable logistic regression models to statistically adjust for its potential confounding effects on metabolic parameters.

#### Machine learning model development

Given significant class imbalance (RA:SjD approximately 8:1), we performed 1:1 random downsampling of the RA majority class to create a balanced training dataset (594 RA + 594 SjD = 1,188 patients). Downsampling was chosen over alternative approaches (e.g., SMOTE, class weighting) for several reasons: (1) it produces a clean, real-data-only training set without synthetic samples; (2) even after downsampling, the training set (*n* = 1,188) retained sufficient statistical power; (3) the large original RA cohort (*n* = 4,736) ensured that the random subsample was representative. To further verify that class imbalance did not bias results, we additionally evaluated model performance on the full unbalanced cohort (*n* = 5,330) through subgroup analyses (Table S6), which showed consistent AUC values across all strata. XGBoost (Extreme Gradient Boosting) algorithm was selected for its robust performance on tabular medical data and built-in feature importance assessment capabilities.^36^ The primary XGBoost model incorporated 14 features: age, sex, ESR, CRP, RF, TC, TG, HDL-C, LDL-C, glucose, ALT, AST, GGT, and GC (binary). HCQ was not included as a feature in the primary model; its confounding effects were separately addressed through multivariable logistic regression with dual GC + HCQ adjustment and HCQ-stratified analyses. In the ablation analysis (Tables S2 and S3), a 15-feature model including HCQ was trained to directly quantify HCQ’s contribution. GC was included as a model feature because of its strong association with SjD treatment patterns (71.9% vs. 13.7% in RA) and its known effects on metabolic parameters including glucose and lipids. In the multivariable logistic regression analysis, GC was included as a covariate to enable estimation of other features’ independent effects after adjusting for GC use.

Disease-specific antibodies including anti-CCP, anti-Ro/SSA, and anti-La/SSB were not included in the model, based on design considerations for specific clinical application scenarios: (1) this model aims to provide an auxiliary diagnostic tool for the clinical gray zone where anti-CCP is negative or indeterminate; (2) we sought to explore pathological metabolic mechanisms independent of ACPA status; (3) when anti-CCP is clearly positive, the diagnosis is usually already relatively clear and does not require additional auxiliary tools.

Hyperparameters were set as follows: maximum tree depth = 4, learning rate (eta) = 0.05, number of boosting rounds = 100, subsample ratio = 0.8, column sampling ratio = 0.8, using binary classification logistic objective function. The balanced dataset was split into training (70%) and test (30%) sets using stratified sampling to ensure complete separation with no data leakage.

#### External validation

The external validation cohort contained missing values in 9 of 14 model features, with missingness ranging from 4.4% (ALT) to 17.6% (RF). Missing data were handled using multiple imputation by chained equations (MICE) with predictive mean matching (pmm) for continuous variables, using 5 imputations and 20 iterations (R package mice, random seed 2026). Performance metrics were pooled across the five MICE-imputed datasets using Rubin’s rules. For AUC, we computed the point estimate and within-imputation standard error (DeLong method) separately for each imputed dataset; the pooled AUC and 95% confidence interval were then obtained by combining the within-imputation and between-imputation variance components according to Rubin’s formulae. The fraction of missing information (λ) was calculated to quantify imputation uncertainty. Brier score and calibration metrics were averaged across imputations. Convergence was verified through trace plots. A complete case sensitivity analysis (*n* = 225, AUC = 0.805, 95% CI: 0.739–0.871) was also performed to assess the impact of imputation on model performance.

The trained XGBoost model from the discovery cohort was directly applied to the independent external validation cohort from the First Affiliated Hospital of Nanchang University without retraining or recalibration. The optimal classification threshold (0.465) was determined using Youden’s J statistic on the discovery cohort’s held-out test set and was applied unchanged (“locked”) to the external validation cohort, with no threshold re-optimization on external data. Model performance was assessed using AUC-ROC, calibration plots, decision curve analysis, and SHAP interpretation. Subgroup analyses stratified by age, sex, RF level, HCQ use, and GC use were performed to evaluate model stability across clinical strata in the external cohort.

#### Transcriptomic corroboration analysis

To explore the molecular basis underlying the clinically observed metabolic differences between RA and SjD, we performed parallel transcriptomic analyses using two publicly available GEO datasets: GSE51092 (primary Sjögren’s syndrome, pSS, *n* = 190 patients vs. *n* = 32 matched healthy controls) and GSE93272 (rheumatoid arthritis, RA, *n* = 232 patients vs. *n* = 43 matched healthy controls). Raw CEL files were processed using the R packages affy and limma. Probes were reannotated using the corresponding annotation packages; probes mapping to multiple genes or lacking gene symbols were removed. Differential expression was assessed using empirical Bayes-moderated linear models, with significance defined as |log2FC| > 1 and adjusted *p* < 0.05 (Benjamini-Hochberg method).

Volcano plots were generated with ggplot2, displaying log2 fold change on the x axis and −log10(adjusted P) on the y axis. Heatmaps were generated using pheatmap, displaying normalized expression values (*Z* score across samples) for the top 20 differentially expressed genes (10 most significantly upregulated and 10 most significantly downregulated) in each disease.

Gene Ontology Biological Process (GO-BP) and Kyoto Encyclopedia of Genes and Genomes (KEGG) pathway enrichment analyses were performed using the clusterProfiler R package, applied to the upregulated DEG sets of each disease. Significantly enriched terms (adjusted *p* < 0.05) were visualized as horizontal dot plots. Importantly, a parallel analysis strategy was adopted—each disease was compared independently against its own matched healthy controls—to avoid batch effects inherent in cross-platform or cross-study comparisons. Pathway-level convergence and divergence were then compared qualitatively between diseases. All statistical analyses were conducted in R (version 4.5.0).

#### Statistical analysis and model evaluation

Baseline characteristics were compared using Wilcoxon rank-sum test (continuous variables) and chi-square test (categorical variables) for the discovery cohort. For the external validation cohort, independent samples *t* test was used for continuous variables, and chi-square or Fisher’s exact test for categorical variables. Model discriminative ability was assessed using area under the receiver operating characteristic curve (AUC-ROC). 95% confidence intervals for AUC were calculated using Bootstrap resampling (*n* = 2,000 iterations). Model stability was evaluated through 5-fold cross-validation, reporting mean AUC and standard deviation.

To compare algorithm performance, we additionally trained logistic regression, random forest, and support vector machine (SVM with RBF kernel) models using the same cross-validation procedure. Model calibration was assessed through calibration plots, Hosmer-Lemeshow goodness-of-fit test, calibration intercept and slope (estimated via logistic recalibration), and Brier score. These metrics were calculated for the internal test set, external validation cohort, and 5-fold cross-validation (Table S4). Model ablation and feature category analyses are described in Tables S2, S3, and S5.

To identify independent discriminating features with interpretable effect sizes, multivariable logistic regression was performed on standardized continuous variables (*Z* score transformation) to enable direct comparison of odds ratios (OR). Forest plots were generated to visualize effect direction and magnitude.

#### Model interpretability analysis

SHAP (SHapley Additive exPlanations) analysis was employed to interpret XGBoost model predictions.^21^ SHAP values quantify each feature’s contribution to individual predictions, providing both local (individual patient) and global (overall) interpretability. We generated SHAP summary plots to visualize feature importance rankings and effect directions, as well as dependence plots for key metabolic features. SHAP analysis was performed independently in both the discovery and external validation cohorts to assess the reproducibility of feature importance rankings.

#### Subgroup and sensitivity analyses

Subgroup analyses were performed by age (≤60 years vs. > 60 years), RF level (≤200 vs. > 200 IU/mL), sex, and diabetes status to assess model stability across clinically relevant strata. Sex-stratified analysis was particularly important because SjD is extremely female-predominant (female:male approximately 9:1 or higher), necessitating validation that the model retains discriminative ability after excluding sex as a factor. Decision curve analysis (DCA) evaluated net clinical benefit across different threshold probabilities.

To exclude potential confounding effects of hepatotoxic medications, sensitivity analysis excluded RA patients receiving methotrexate or leflunomide treatment. In this sensitivity analysis, multivariable logistic regression was adjusted for glucocorticoid use to control for potential confounding effects of corticosteroids on metabolic parameters.

To validate model effectiveness in the target application population of “serologically atypical” patients, we performed independent validation analysis in the anti-CCP-negative RA subgroup. This subgroup included 163 ACPA-negative RA patients compared with 594 SjD-Phenotype 1 patients.

Additionally, to address the potential confounding effect of hydroxychloroquine (HCQ) on metabolic profiles, we performed three complementary analyses. First, we constructed a multivariable logistic regression model incorporating both GC and HCQ simultaneously as covariates. Second, we performed HCQ-stratified analyses comparing metabolic parameters between RA and SjD-Phenotype 1 separately within HCQ users and non-users. Third, we evaluated the XGBoost model’s discriminative performance (AUC) separately in HCQ users and non-users.

### Quantification and statistical analysis

#### Statistical analysis

All analyses were performed using R version 4.5.0, with packages including xgboost, caret, pROC, SHAPforxgboost, ggplot2, limma, and clusterProfiler. Statistical significance was defined as *p* < 0.05 (two-sided). Given the multiple comparisons involved in baseline characteristic comparisons, we applied Bonferroni correction to the 17 variables in Table 1, with an adjusted significance threshold of *p* < 0.05/17 = 0.00294. The study flowchart is shown in Figure S3.

In Table 1, asterisks (∗) denote statistical significance after Bonferroni correction for multiple comparisons (adjusted threshold: *p* < 0.05/17 = 0.00294). Wilcoxon rank-sum test was used for continuous variables and chi-square test for categorical variables.

## Supplemental information

Document S1. Figures S1–S5

Table S1. Hydroxychloroquine (HCQ) confounding analysis, related to Figure 2 and STAR Methods

Table S2. Model ablation analysis, related to Figure S4

Table S3. Feature category model comparison, related to Figure S4

Table S4. Calibration metrics, related to Figure S5

Table S5. Treatment confounding sensitivity analyses, related to Figure S5

Table S6. Exploratory subgroup model performance of discovery cohort, related to Table 2 and STAR Methods
