# Supplementary material for: Clinical-metabolic machine learning model differentiating rheumatoid arthritis from high-inflammatory Sjögren disease: A multi-center study
Source: iScience. 2026 Aug 24;29(9):117299. doi: 10.1016/j.isci.2026.117299 (PMC13542512; doi:10.1016/j.isci.2026.117299)
Supplement: Supplementary file 1 [file mmc1.pdf]

iScience, Volume *29*

Supplemental information

Clinical-metabolic machine learning model differentiating rheumatoid arthritis from high-inflammatory Sjögren disease: A multi-center study

Jianbin Li, Suiran Li, Renhe Li, Ning Tan, Xiaoqing Wang, Mengxia Liu, Yuzhen Gesang, and Wei Liu

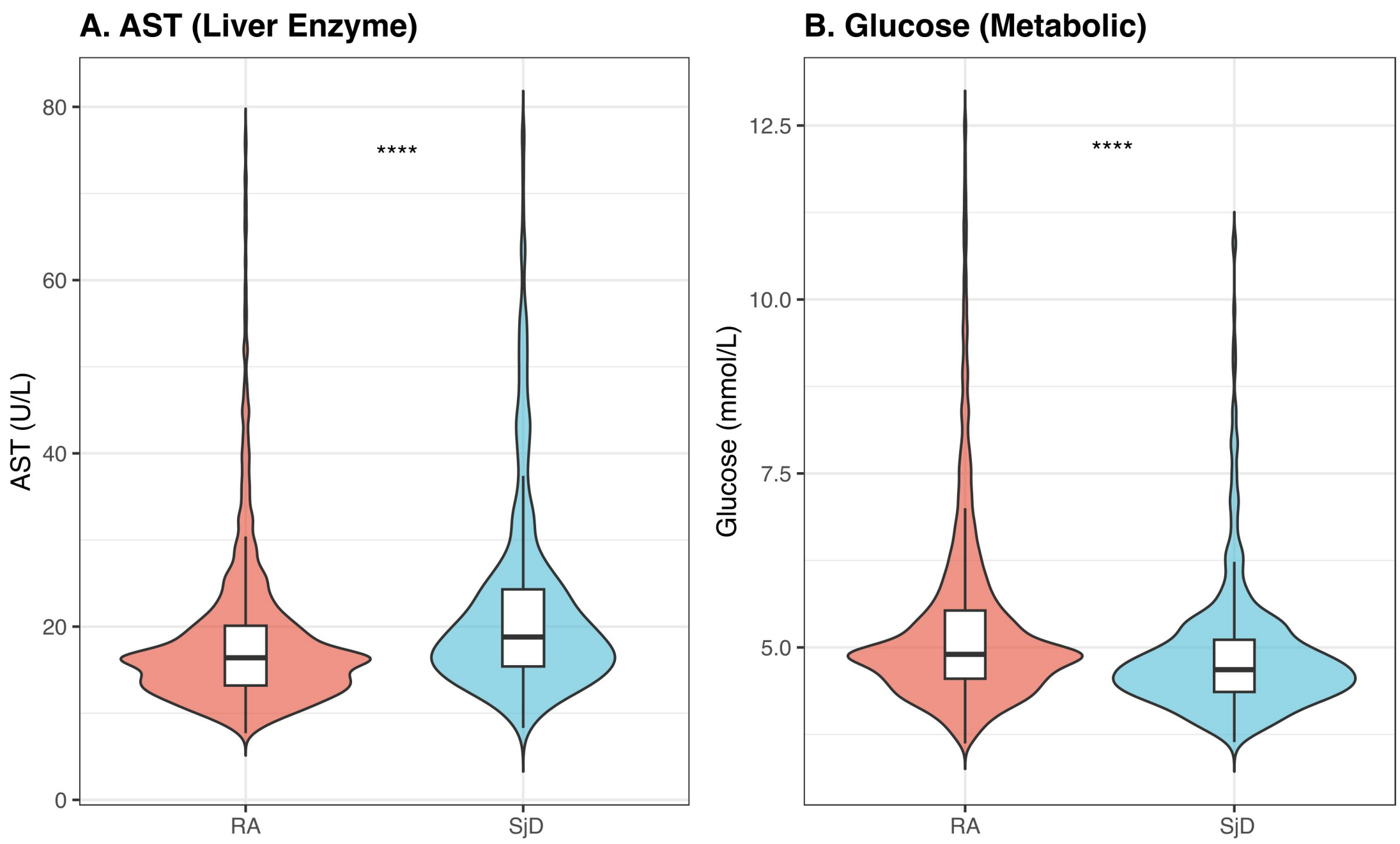

Figure S1. Distribution of key metabolic features between RA and SjD-Phenotype 1 groups. Violin plots with embedded box plots showing the distribution of (A) AST (U/L) and (B) fasting glucose (mmol/L) between RA and SjD-Phenotype 1 patients. The width of the violin represents data density; the inner box plot shows the median (horizontal line), interquartile range (box), and data range (whiskers). Statistical significance was assessed using the Wilcoxon rank-sum test (****$p < 0.0001$). RA patients exhibited significantly higher glucose levels, while SjD-Phenotype 1 patients exhibited significantly higher AST levels, consistent with the distinct metabolic profiles identified by the model.

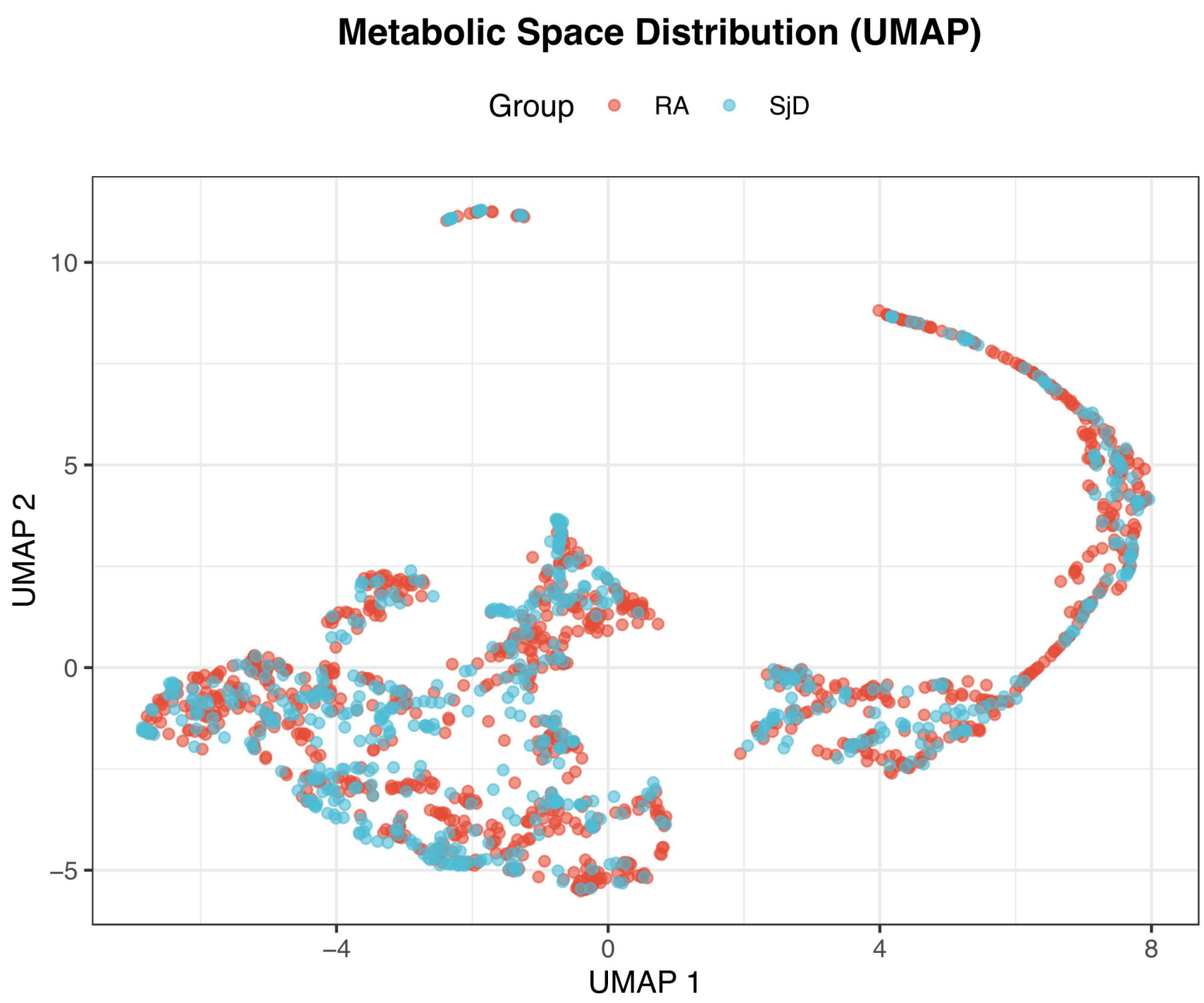

Figure S2. UMAP dimensionality reduction visualization of the metabolic feature space. Uniform Manifold Approximation and Projection (UMAP) plot showing the distribution of RA (red) and SjD-Phenotype 1 (blue) patients in the two-dimensional space derived from 14 model features (including GC as a binary variable). Partial separation between the two groups is visible, indicating that the clinical-metabolic feature space captures meaningful inter-group differences. The overlap region corresponds to the diagnostically challenging cases where the model provides auxiliary discriminative value.

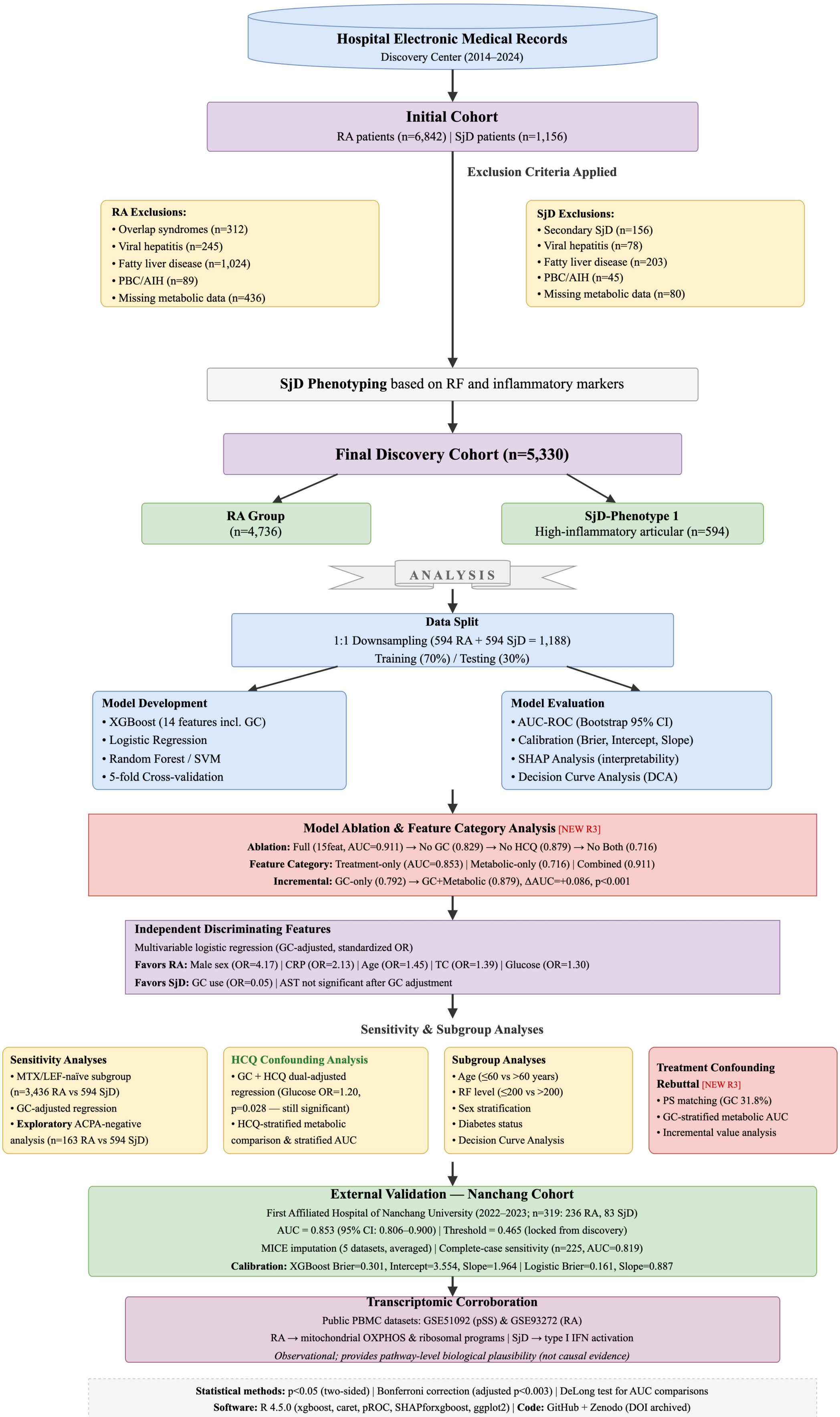
Hospital Electronic Medical Records
Discovery Center (2014–2024)
Initial Cohort
RA patients (n=6,842) | SjD patients (n=1,156)
Exclusion Criteria Applied
RA Exclusions:
• Overlap syndromes (n=312)
• Viral hepatitis (n=245)
• Fatty liver disease (n=1,024)
• PBC/AIH (n=89)
• Missing metabolic data (n=436)
SjD Exclusions:
• Secondary SjD (n=156)
• Viral hepatitis (n=78)
• Fatty liver disease (n=203)
• PBC/AIH (n=45)
• Missing metabolic data (n=80)
SjD Phenotyping based on RF and inflammatory markers
Final Discovery Cohort (n=5,330)
RA Group
(n=4,736)
SjD-Phenotype 1
High-inflammatory articular (n=594)
ANALYSIS
Data Split
1:1 Downsampling (594 RA + 594 SjD = 1,188)
Training (70%) / Testing (30%)
Model Development
• XGBoost (14 features incl. GC)
• Logistic Regression
• Random Forest / SVM
• 5-fold Cross-validation
Model Evaluation
• AUC-ROC (Bootstrap 95% CI)
• Calibration (Brier, Intercept, Slope)
• SHAP Analysis (interpretability)
• Decision Curve Analysis (DCA)
Model Ablation & Feature Category Analysis [NEW R3]
Ablation: Full (15feat, AUC=0.911) → No GC (0.829) → No HCQ (0.879) → No Both (0.716)
Feature Category: Treatment-only (AUC=0.853) | Metabolic-only (0.716) | Combined (0.911)
Incremental: GC-only (0.792) → GC+Metabolic (0.879), ΔAUC=+0.086, p<0.001
Independent Discriminating Features
Multivariable logistic regression (GC-adjusted, standardized OR)
Favors RA: Male sex (OR=4.17) | CRP (OR=2.13) | Age (OR=1.45) | TC (OR=1.39) | Glucose (OR=1.30)
Favors SjD: GC use (OR=0.05) | AST not significant after GC adjustment
Sensitivity & Subgroup Analyses
Sensitivity Analyses
• MTX/LEF-naïve subgroup (n=3,436 RA vs 594 SjD)
• GC-adjusted regression
• Exploratory ACPA-negative analysis (n=163 RA vs 594 SjD)
HCQ Confounding Analysis
• GC + HCQ dual-adjusted regression (Glucose OR=1.20, p=0.028 — still significant)
• HCQ-stratified metabolic comparison & stratified AUC
Subgroup Analyses
• Age (≤60 vs >60 years)
• RF level (≤200 vs >200)
• Sex stratification
• Diabetes status
• Decision Curve Analysis
Treatment Confounding Rebuttal [NEW R3]
• PS matching (GC 31.8%)
• GC-stratified metabolic AUC
• Incremental value analysis
External Validation — Nanchang Cohort
First Affiliated Hospital of Nanchang University (2022–2023; n=319: 236 RA, 83 SjD)
AUC = 0.853 (95% CI: 0.806–0.900) | Threshold = 0.465 (locked from discovery)
MICE imputation (5 datasets, averaged) | Complete-case sensitivity (n=225, AUC=0.819)
Calibration: XGBoost Brier=0.301, Intercept=3.554, Slope=1.964 | Logistic Brier=0.161, Slope=0.887
Transcriptomic Corroboration
Public PBMC datasets: GSE51092 (pSS) & GSE93272 (RA)
RA → mitochondrial OXPHOS & ribosomal programs | SjD → type I IFN activation
Observational; provides pathway-level biological plausibility (not causal evidence)
Statistical methods: p<0.05 (two-sided) | Bonferroni correction (adjusted p<0.003) | DeLong test for AUC comparisons
Software: R 4.5.0 (xgboost, caret, pROC, SHAPforxgboost, ggplot2) | Code: GitHub + Zenodo (DOI archived)

Figure S3. Study design and analytical workflow. Patients were screened from the hospital electronic medical record system of the discovery center (2014–2024). After excluding overlap syndromes or secondary Sjögren disease, viral hepatitis, non-alcoholic fatty liver disease, PBC/AIH, history of chronic heavy alcohol consumption, and cases with missing key variables, 4,736 RA patients and 594 SjD-Phenotype 1 (high-inflammatory articular phenotype) patients were included in the discovery cohort (n=5,330). Given the class imbalance (approximately 8:1), 1:1 random downsampling was performed to create a balanced dataset (594 RA + 594 SjD = 1,188), which was split into training (70%) and test (30%) sets. Discriminative models were constructed using XGBoost (incorporating 14 features including GC as a binary variable), logistic regression, random forest, and SVM, with 5-fold cross-validation. Model evaluation included AUC-ROC, calibration assessment (Brier score, calibration intercept and slope, Hosmer-Lemeshow test), SHAP interpretability analysis, and decision curve analysis. Model ablation analysis systematically assessed the contribution of treatment variables by training models excluding GC, HCQ, or both; feature category analysis compared treatment-only, metabolic-only, and combined models, with incremental value analysis quantifying the independent contribution of metabolic features beyond treatment patterns (Tables S2–S3, Figure S4). Treatment confounding was further addressed through propensity score matching on GC use, GC-stratified metabolic model evaluation, and incremental value analysis (Table S5). Multivariable logistic regression (GC-adjusted) identified independent discriminating features. Sensitivity analyses included: subgroup analysis excluding methotrexate/leflunomide (n=3,436 RA vs 594 SjD), GC-adjusted regression, exploratory ACPA-negative subgroup analysis (n=163 RA vs 594 SjD; includes training samples), and comprehensive hydroxychloroquine (HCQ) confounding evaluations (GC+HCQ dual-adjusted regression, HCQ-stratified comparisons, and stratified modeling). Subgroup analyses assessed model stability across age, RF level, sex, and diabetes strata. External validation was independently conducted without retraining or recalibration on a geographically distinct cohort from the First Affiliated Hospital of Nanchang University (2022–2023; n=319: 236 RA, 83 SjD), with comprehensive calibration assessment (Table S4, Figure S5). The classification threshold (0.465) was determined using Youden's J statistic on the discovery test set and applied unchanged to the external cohort. Parallel transcriptomic analyses of public PBMC datasets (GSE51092 and GSE93272) were performed to

provide molecular-level corroborative evidence for the clinically observed metabolic divergence; these observational analyses provide biological plausibility but do not establish causal relationships. All analysis code and model specifications are publicly archived at GitHub with a persistent DOI via Zenodo. Statistical significance was defined as $p<0.05$ (two-sided) with Bonferroni correction for multiple comparisons (adjusted threshold $p<0.00294$) and DeLong tests for AUC comparisons.

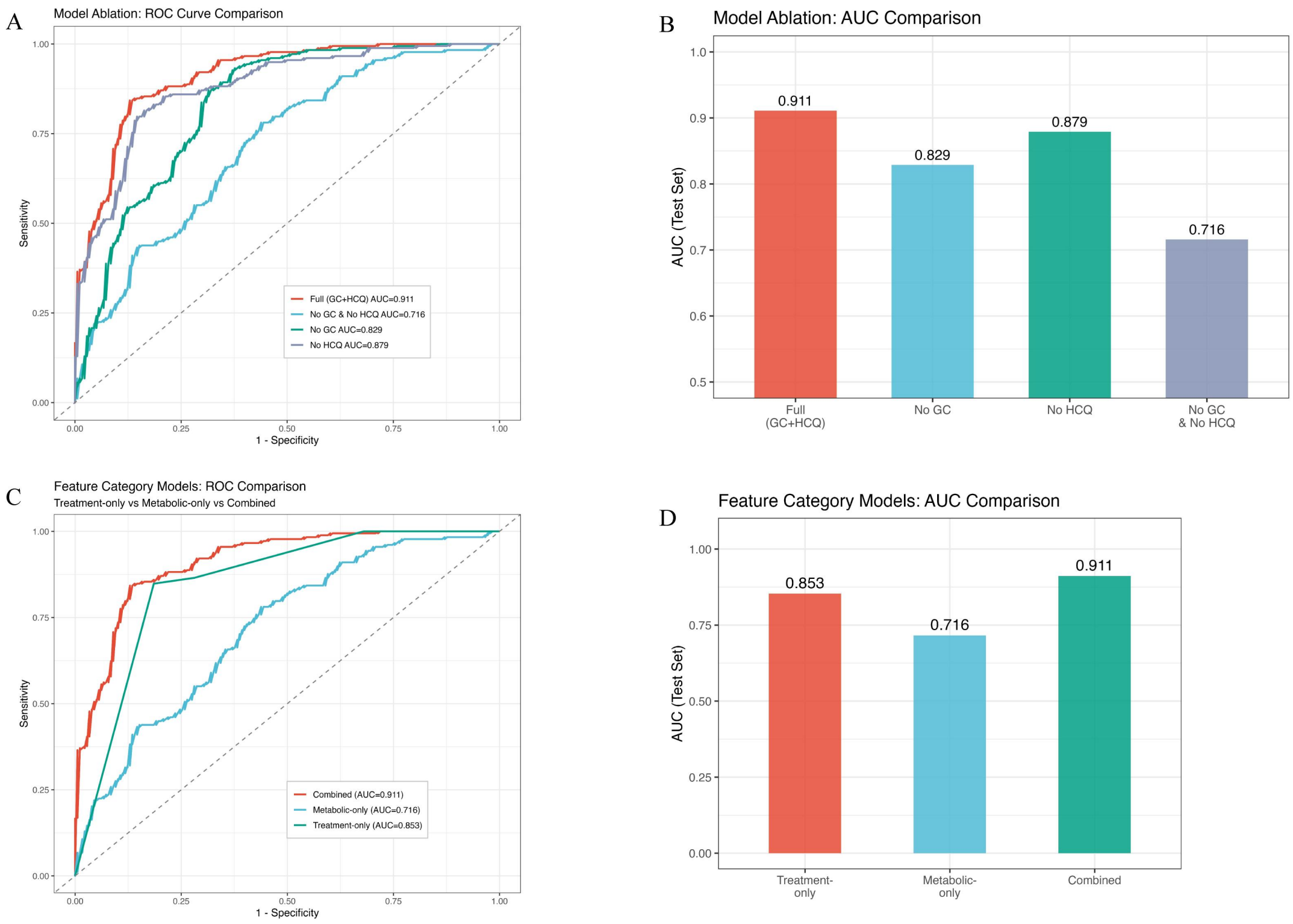

Figure S4. Model ablation and feature category analysis. (A) ROC curves comparing four ablation models on the held-out test set: Full model (GC+HCQ, 15 features; AUC = 0.911), No GC model (14 features; AUC = 0.829), No HCQ model (14 features; AUC = 0.879), and No GC & No HCQ model (13 features; AUC = 0.716). The dashed diagonal line represents random classification (AUC = 0.5).

(B) Bar chart comparing AUC values across the four ablation models, demonstrating the progressive loss of discriminative ability upon removal of treatment variables. (C) ROC curves comparing three feature category models: Treatment-only (GC+HCQ, 2 features; AUC = 0.853), Metabolic-only (13 clinical features; AUC = 0.716), and Combined (all 15 features; AUC = 0.911). All pairwise comparisons were statistically significant (DeLong $p < 0.001$).

(D) Bar chart comparing AUC values across the three feature category models, illustrating that the

combined model significantly outperforms both individual category models. GC, glucocorticoid; HCQ, hydroxychloroquine; AUC, area under the receiver operating characteristic curve.

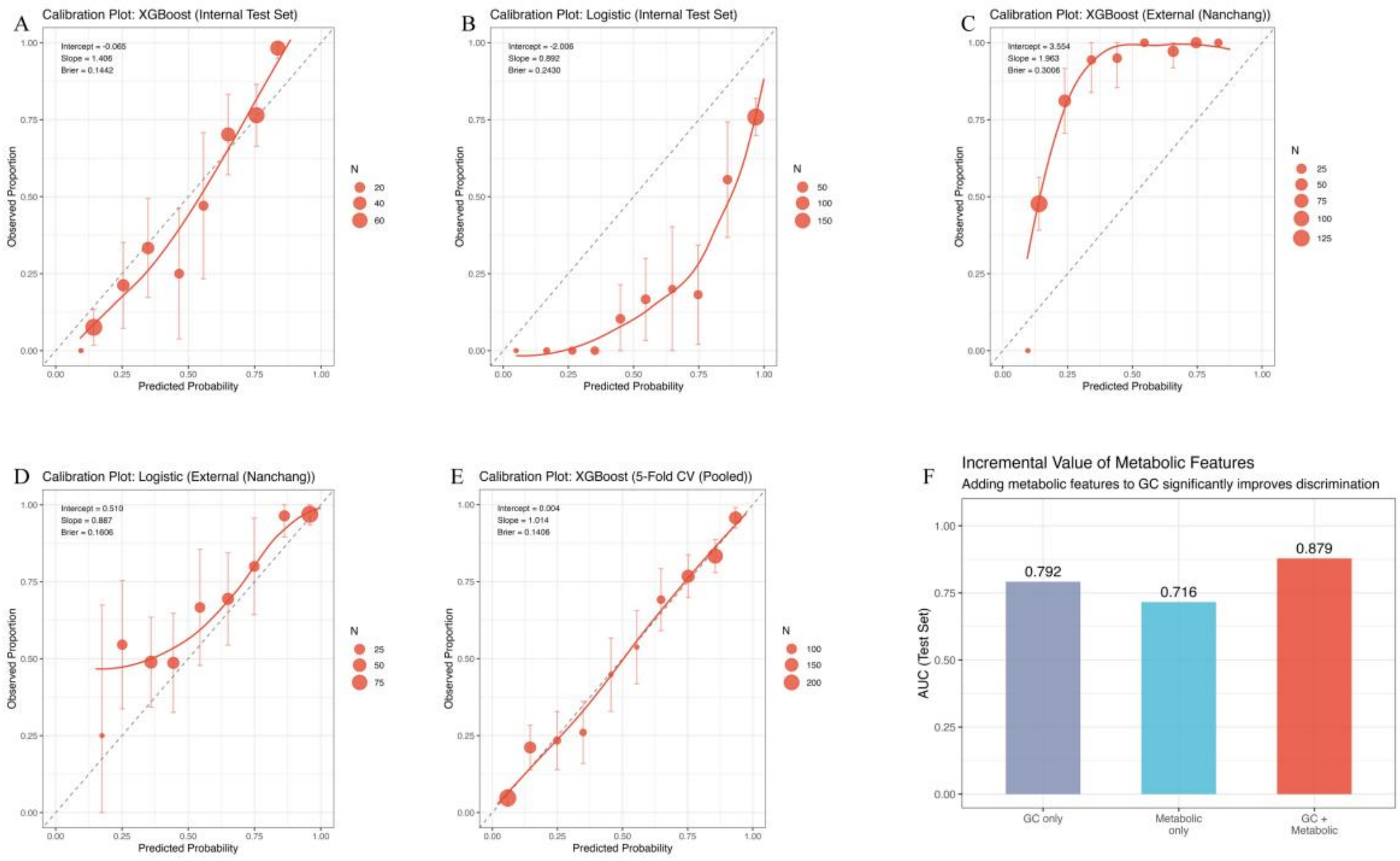

Figure S5. Calibration assessment and incremental value analysis. (A) Calibration plot for the XGBoost model on the internal test set (Brier = 0.144, intercept = −0.065, slope = 1.406). (B) Calibration plot for the logistic regression model on the internal test set (Brier = 0.243, intercept = −2.006, slope = 0.892). (C) Calibration plot for the XGBoost model on the external validation cohort (Nanchang; pooled Brier = 0.195, intercept = 1.409, slope = 1.225; averaged across 5 MICE imputations), showing calibration deviation expected in a geographically distinct population.

(D) Calibration plot for the logistic regression model on the external validation cohort (pooled Brier = 0.207), demonstrating better external calibration compared to XGBoost. (E) Pooled calibration plot from 5-fold cross-validation for the XGBoost model (Brier = 0.141, intercept = 0.004, slope = 1.014), confirming good internal calibration. In panels A–E, the dashed diagonal line represents perfect calibration; dot sizes are proportional to the number of observations per decile group; error bars represent 95% confidence intervals.

(F) Incremental value analysis bar chart showing AUC improvement when adding metabolic features to GC alone: GC-only (AUC = 0.792) → Metabolic-only (AUC = 0.716) → GC + Metabolic (AUC = 0.879), with a ΔAUC of 0.086 (DeLong $p < 0.001$) from GC-only to GC + Metabolic, demonstrating independent metabolic discriminative contribution.
